# Supplementary material for: Epigenetic reprogramming shapes the cellular landscape of schwannoma
Source: Nat Commun. 2024 Jan 12;15:476. doi: 10.1038/s41467-023-40408-5 (PMC10786948; doi:10.1038/s41467-023-40408-5)
Supplement: Supplementary file 1 — Supplementary Information [file 41467_2023_40408_MOESM1_ESM.pdf]

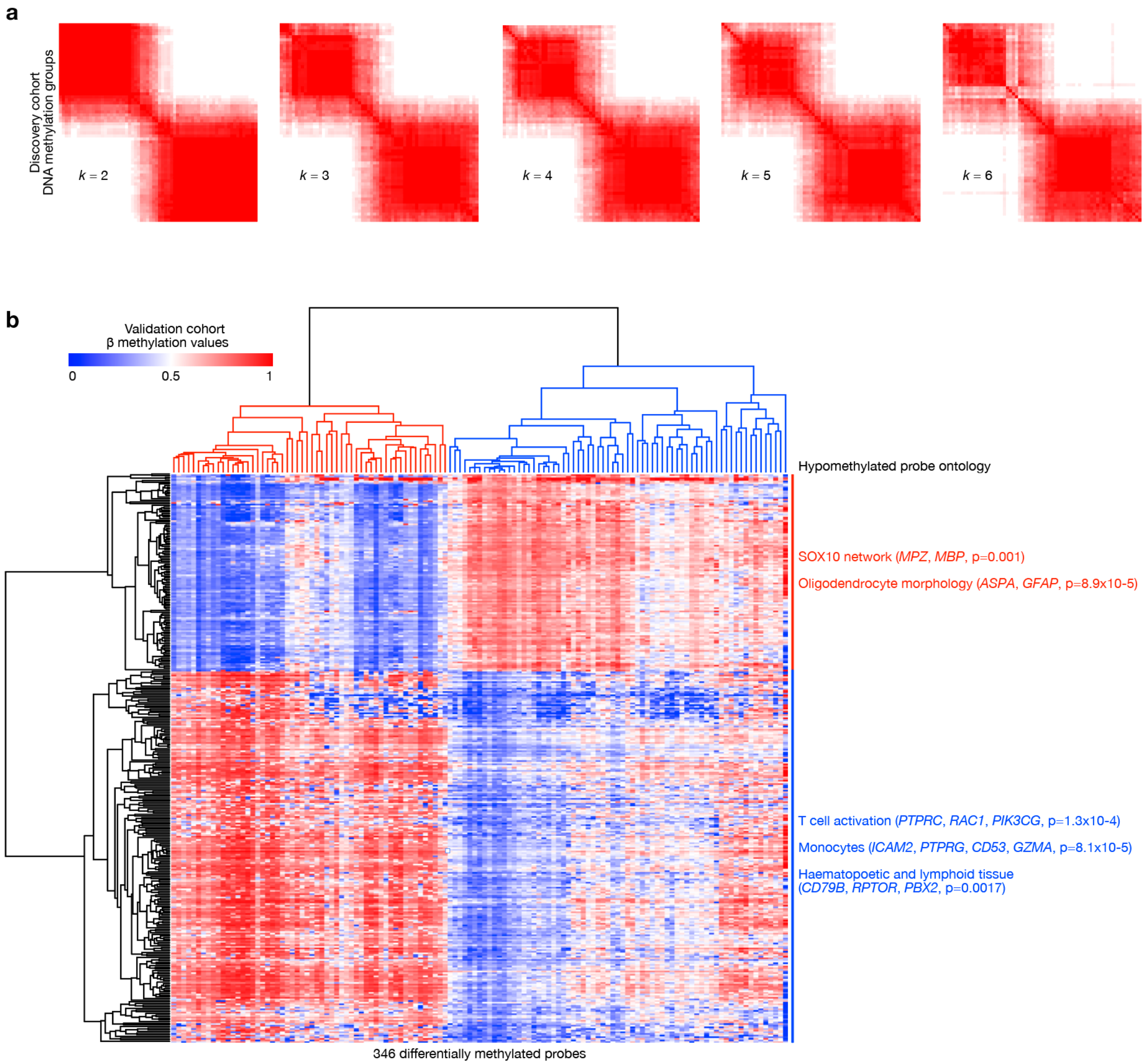

Supplementary Fig. 1. DNA methylation-based classification of schwannomas identifies 2 molecular groups. a, Consensus clustering of pairwise Pearson correlation coefficients from 850k DNA methylation profiling of 66 vestibular schwannomas (k=2-6) comprising the discovery cohort from UCSF. **b**, Hierarchical clustering of 450K DNA methylation profiles from 125 vestibular or spinal schwannomas comprising the validation cohort[1] using DNA methylation probes overlapping with the top 2000 differentially methylated probes from the discovery cohort (346 probes, Fig. 1a). Significant gene ontology terms of hypomethylated probes distinguishing molecular groups are shown, validating neural crest schwannomas (NCS) and immune-enriched schwannomas (IES). Source data are provided as a Source Data file.

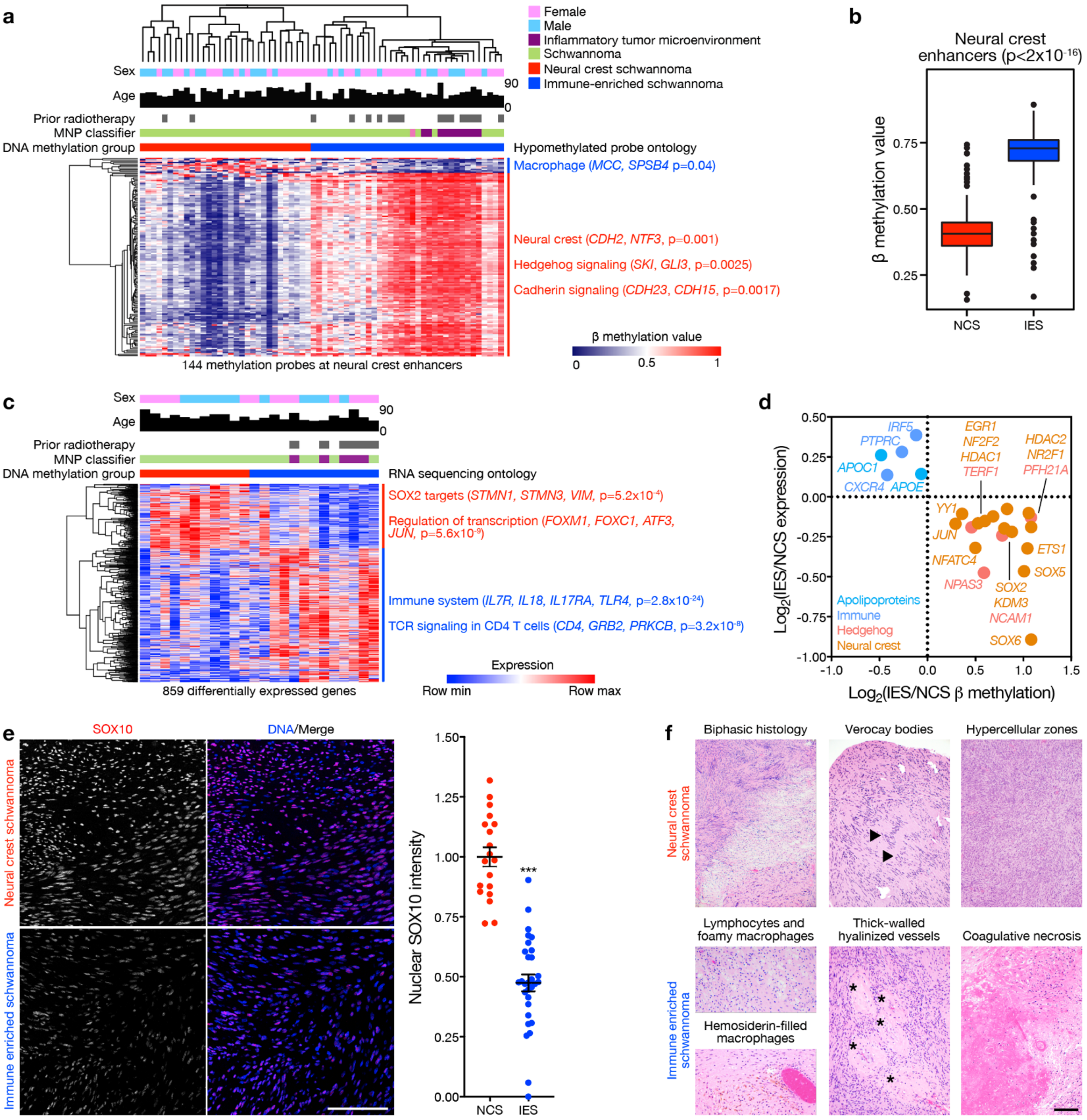

Supplementary Fig. 2. Epigenetic, transcriptomic, protein expression, and histologic validation of neural crest and immune enriched schwannomas. a, Hierarchical clustering of schwannomas (n=66) according to DNA methylation probes overlapping with neural crest enhancers. Significant gene ontology terms corresponding to hypomethylated probes in neural crest schwannomas (NCS) or immune-enriched schwannomas (IES), clinical metadata, and the molecular neuropathology (MNP) DNA methylation classification of central nervous system tumors[2] are shown. **b**, Distribution of median schwannoma β methylation values for DNA methylation probes overlapping with neural crest enhancers. P-value determined using two-sided Kolmogorov-Smirnov test. Boxplots show 1st quartile, median, and 3rd quartile. Whiskers represent 1.5 inter-quartile range, and data outside range are shown (n=31 NCS, n=35 IES). **c**, Hierarchical clustering of differentially expressed genes from RNA sequencing of NCS or IES (n=24). Significant gene ontology terms corresponding to enriched genes in each molecular group and meta-data are shown as in **a**. **d**, Select schwannoma apolipoprotein, immune, Hedgehog, and neural crest genes with anti-correlated expression and β methylation from 24 tumors with matched RNA

sequencing and DNA methylation profiling. **e**, Quantitative immunofluorescence microscopy for the schwannoma differentiation marker SOX10 across NCS or IES (n=49 schwannomas). Scale bar, 100μm. Lines represent means and error bars represent standard error of means (Two-sided Student's t test, ***$p=1.12 \times 10^{-12}$). **f**, H&E-stained sections of schwannomas (n=66) showing biphasic histology (90% versus 67%, p=0.0001), Verocay bodies (arrows, 84% vs 47%, p<0.0001) and hypercellular zones (30% vs 3%, p=0.0001) in NCS versus lymphocyte and macrophage infiltration (59% versus 17%, p<0.0001), hemosiderin deposition (93% versus 68%, p<0.0001), hyalinized or thick-walled vessels (asterisks, 74% versus 57%, p=0.017), and necrosis (18% versus 3%, p=0.0008) in IES. Two-sided Fisher's exact tests. Scale bar, 100μm. Source data are provided as a Source Data file.

a

Schwannoma	Sequencing	Prior radiotherapy	C0	C1	C2	C3	C4	C5	C6	C7	C8	C9	C10	C11	C12	Total
NCS1	Single-cell	0	2	20	12	44	9	10	4	13	7	8	14	7	7	157
NCS2	Single-nuclei	0	18	10	14	5	8	2	9	145	5	2	3	0	10	231
NCS3	Single-nuclei	0	72	162	21	104	18	45	16	45	13	50	11	7	32	596
NCS4	Single-nuclei	0	97	167	14	294	19	14	193	34	122	16	2	16	8	996
IES1	Single-cell	0	31	79	424	109	393	47	32	26	85	61	33	34	28	1382
IES2	Single-cell	0	5	41	201	5	122	27	21	94	166	11	20	23	18	754
IES3	Single-nuclei	1	82	53	50	4	5	2	110	37	20	4	7	2	4	380
IES4	Single-nuclei	1	205	1667	35	292	33	142	76	12	21	175	62	57	27	2804
IES5	Single-nuclei	0	2235	413	101	6	45	337	7	54	9	90	14	2	14	3327
Total			2747	2612	872	863	652	626	468	460	448	417	166	148	148	10628

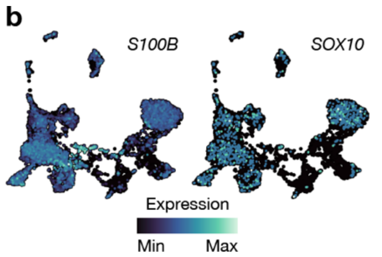

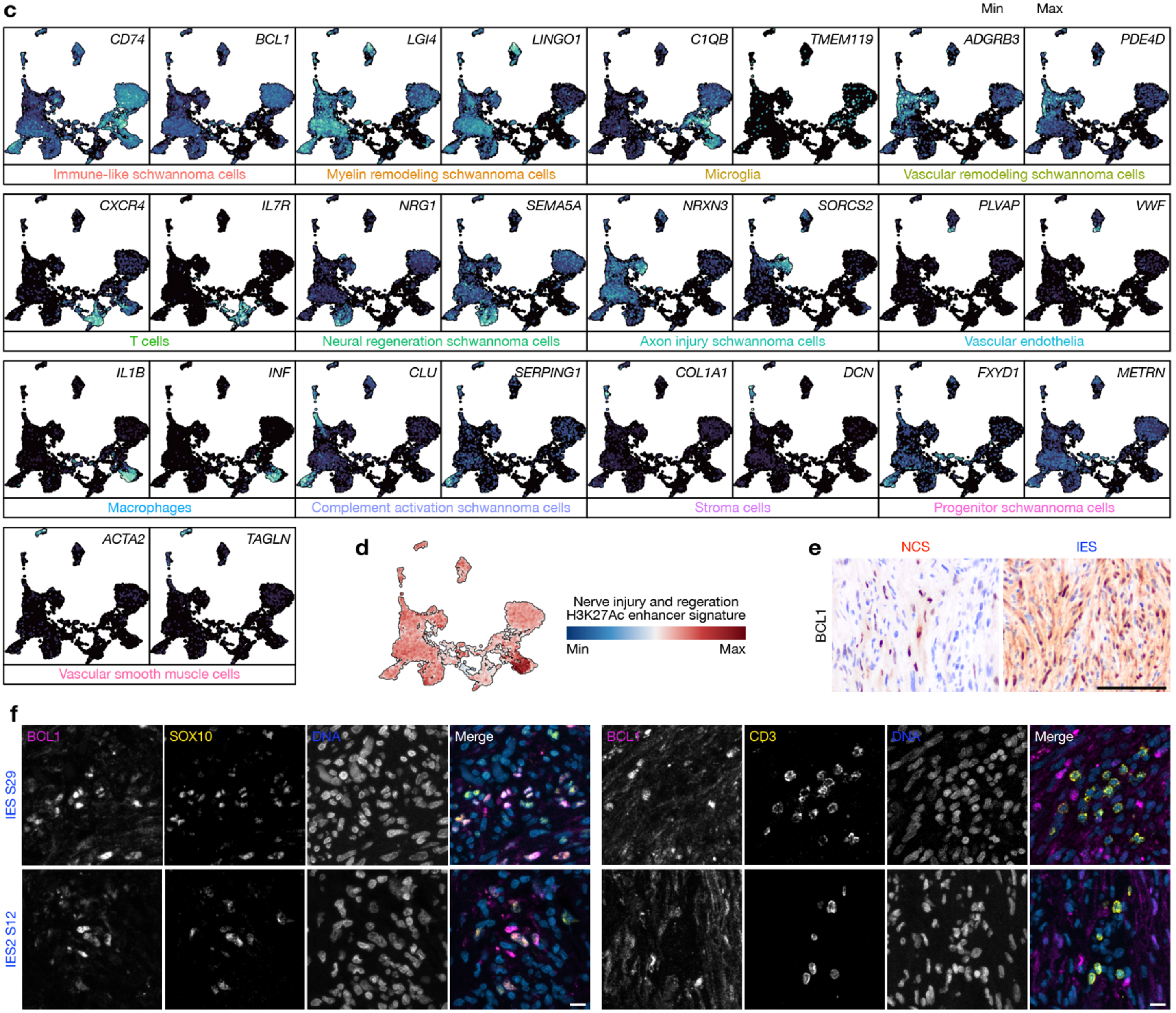

Supplementary Fig. 3. Integrated schwannoma single-nuclei and single-cell RNA sequencing. a, Distribution of integrated, harmonized schwannoma single-nuclei (n=6) and single-cell RNA sequencing (n=3) transcriptomes (Fig. 1b). **b**, Feature plot of integrated UMAP from harmonized schwannoma single-nuclei and single-cell RNA sequencing showing expression of the schwannoma differentiation marker genes *S100B* (left) or *SOX10* (right). **c**, Feature plots of schwannoma and microenvironment cell type marker genes. Immune-like schwannoma cells were distinguished by enrichment of *CD74*, which mediates macrophage migration, axon repair, and survival of neural progenitor cells[3,4]. Myelin remodeling schwannoma cells were distinguished by enrichment of *LINGO1*, which regulates myelin recovery in demyelinating disease and contributes to nerve regeneration[5,6]. Vascular remodeling schwannoma cells were distinguished by enrichment of *ADGRB3*, an angiogenesis regulator that contributes to cell proliferation through the tumor microenvironment[7–9], and the balance of angiogenesis factors is a hallmark of schwannoma[10]. Neural regeneration schwannoma cells were distinguished by enrichment of *SEMA5A*, an inhibitory axon guidance molecule that facilitates schwannoma cell proliferation by modulating axon growth[11,12]. Axon injury schwannoma cells were distinguished by enrichment of

SORCS2, which is induced in peripheral nerve injury[13], and *NRXN3*, which localizes to injured axons[14]. Complement pathway activation schwannoma cells were distinguished by enrichment of *CLU* and *SERPING1*, which are activated in both ischemic and traumatic brain injury[15–17]. Progenitor schwannoma cells were distinguished by enrichment of *FXYD1* and *METRN*, which are expressed in adult neural stem cells[18]. **d**, Mean expression signature of genes neighboring enhancers that are activated during nerve injury and regeneration in integrated schwannoma transcriptomes[19]. **e**, IHC for the immune-like schwannoma cell marker BCL1 reveals enrichment in IES compared to NCS (Two-sided Fisher's exact test, p=0.05). Scale bar, 100µm. Similar results were obtained for *** other samples. **f**, Co-immunofluorescence microscopy for the immune-like schwannoma cell marker BCL1 with the T cell marker CD3 (right) or the schwannoma differentiation marker SOX10 (left) in IES, demonstrating BCL1 expression from schwannoma cells but not from lymphocytes, in support of single-nuclei and single-cell RNA sequencing data (Extended Data Fig. 3c). Scale bar, 10µm. Similar results were obtained for 4 other samples. Source data are provided as a Source Data file.

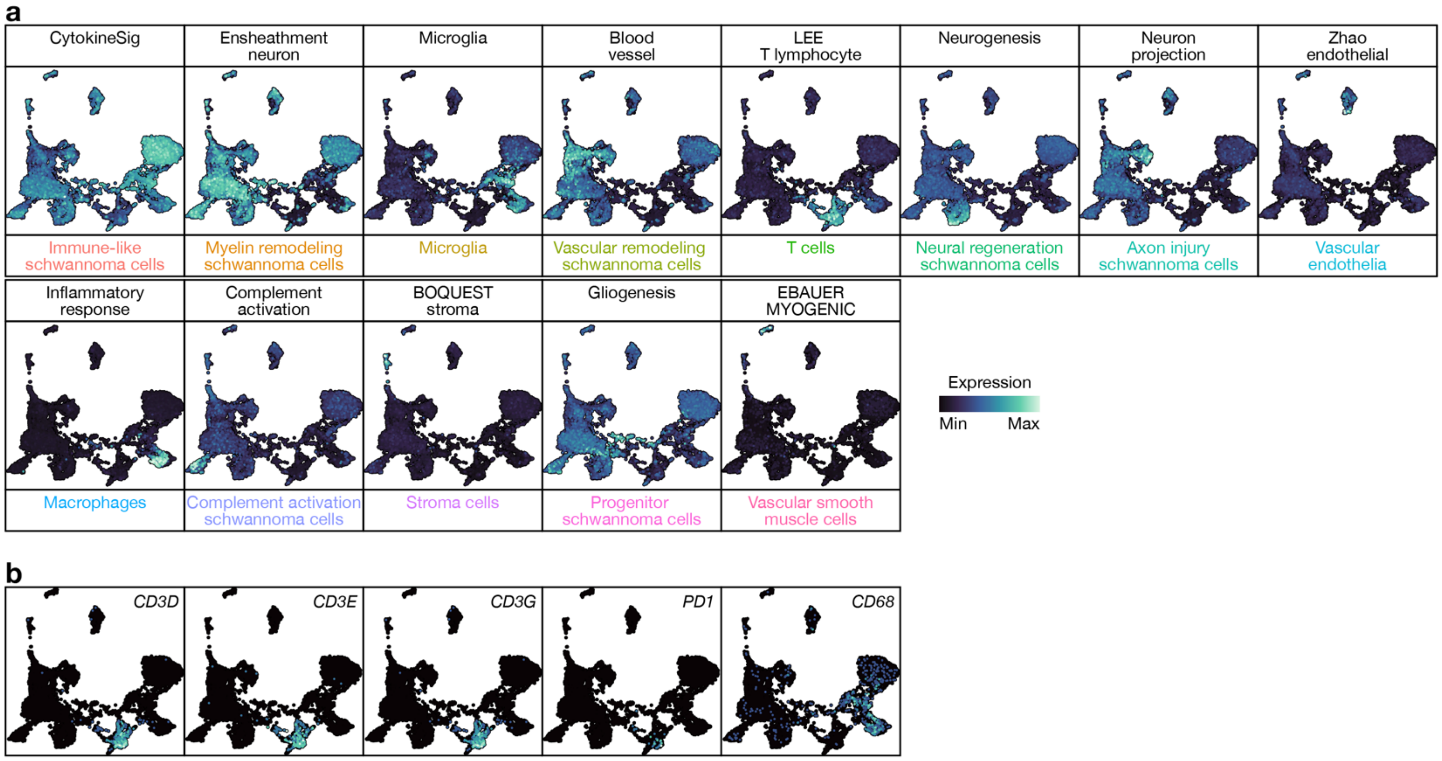

Supplementary Fig. 4. Integrated schwannoma single-nuclei and single-cell RNA sequencing feature plots. a, Normalized gene expression feature plots of signatures derived from Molecular Signatures Database[20] gene sets intersecting with top cluster markers from integrated single-nuclei and single-cell RNA sequencing of human schwannomas supporting schwannoma cell type or microenvironment cell type definitions. **b**, Gene expression feature plots of lymphoid or myeloid marker genes supporting immune cell type definitions. Related to Fig. 1b. Source data are provided as a Source Data file.

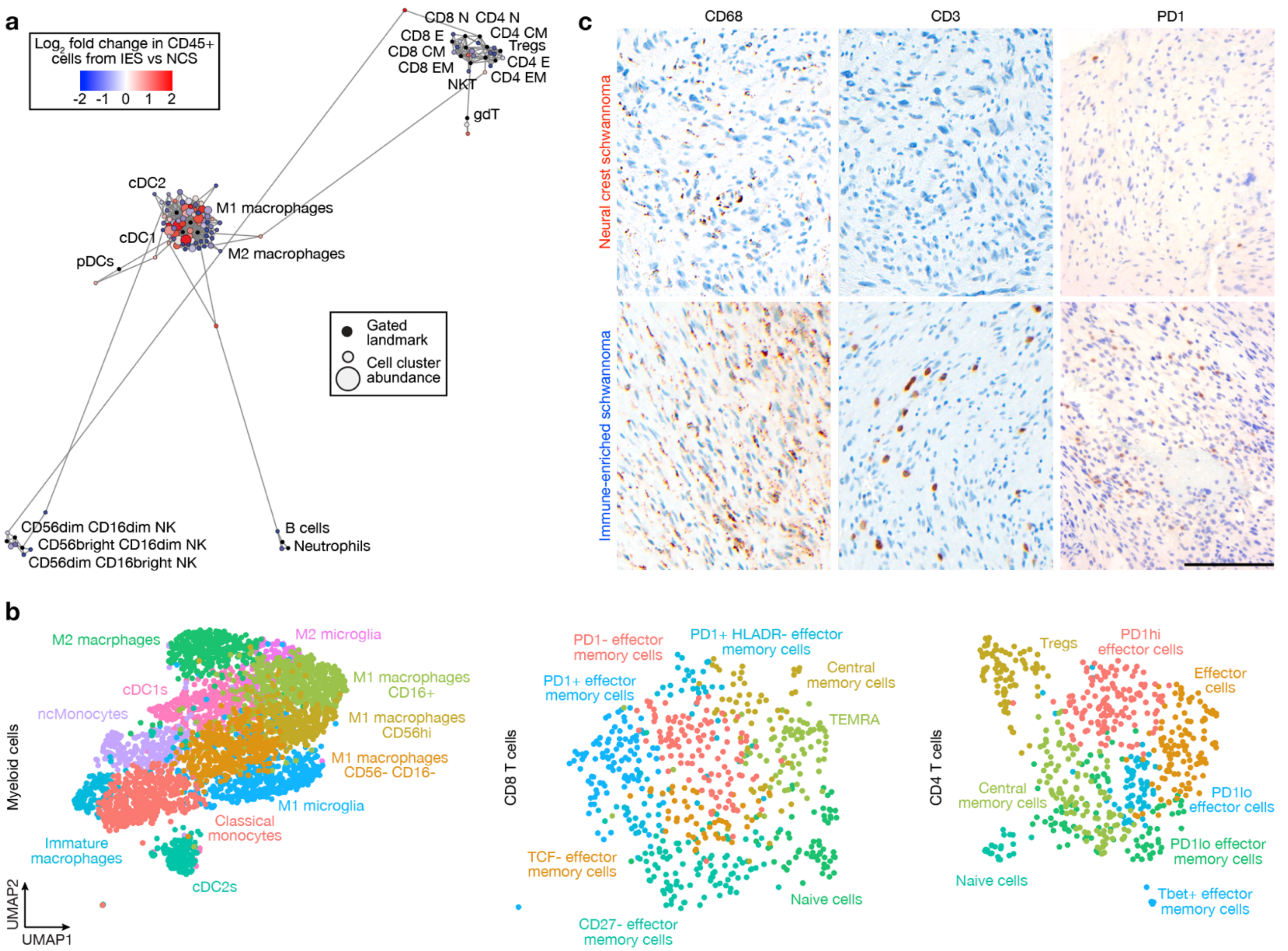

Supplementary Fig. 5. Neural crest and immune enriched schwannomas are distinguished by myeloid and CD8 T cell populations. a, Scaffold plot comprised of 375,355 immune cells from NCS (n=3) or IES (n=3) analyzed using mass cytometry time-of-flight (CyTOF). Manually gated landmark immune cell populations (black) are annotated. **b**, UMAP representation of myeloid cells, CD8 T cells, and CD4 T cells from schwannomas analyzed using CyTOF. **c**, Immunohistochemistry staining for CD68 macrophages (p=0.05), CD3 T cells (p=0.04), and PD1 T cells (p=0.008) reveals enrichment in IES relative to NCS (n=66). Two-sided Fisher's exact tests. Scale bar, 100µm. Source data are provided as a Source Data file.

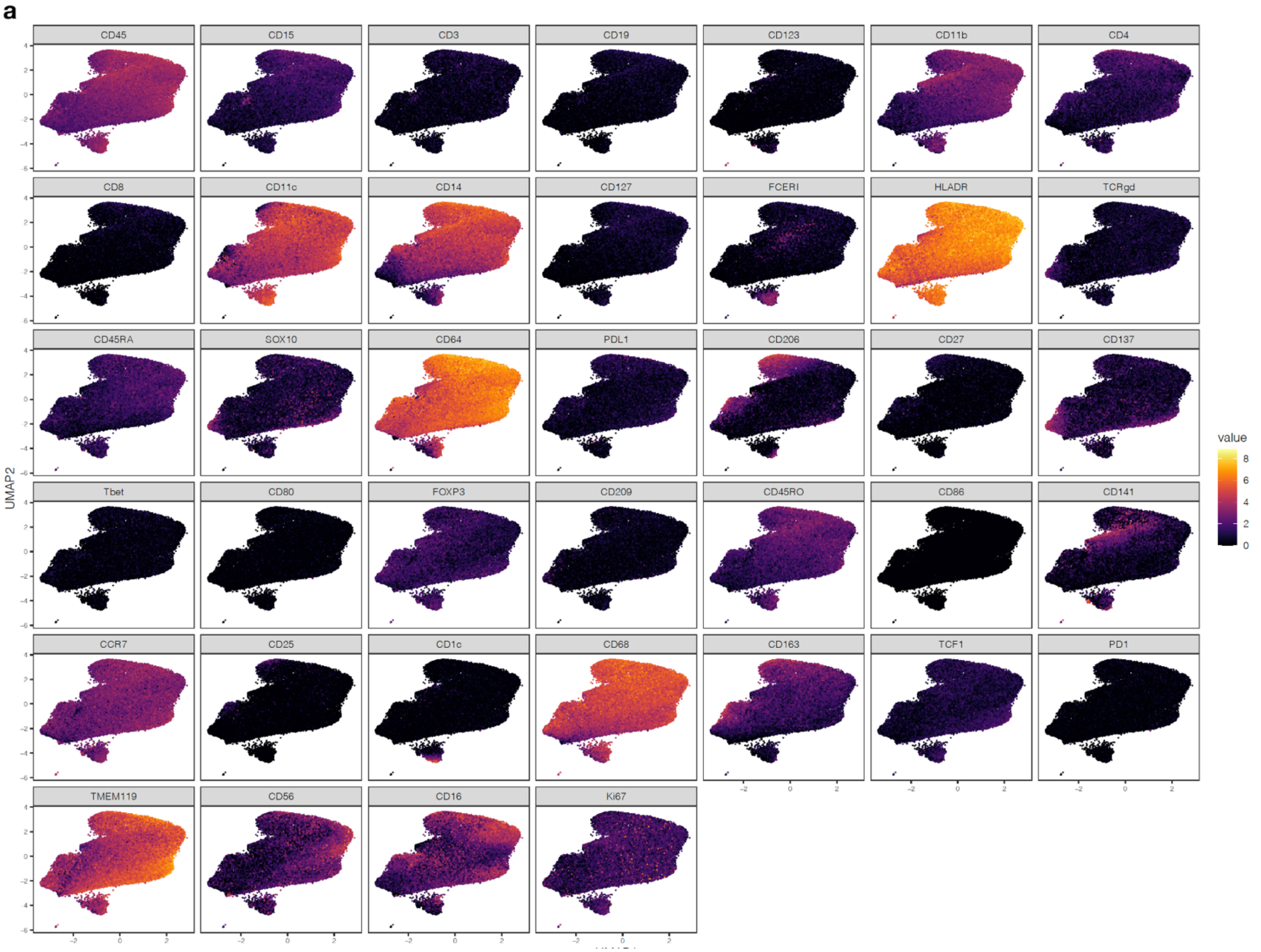

Supplementary Fig. 6. CyTOF myeloid cell feature plots. Related to Fig. 1f, g. Legend shows asinh intensity. Source data are provided as a Source Data file.

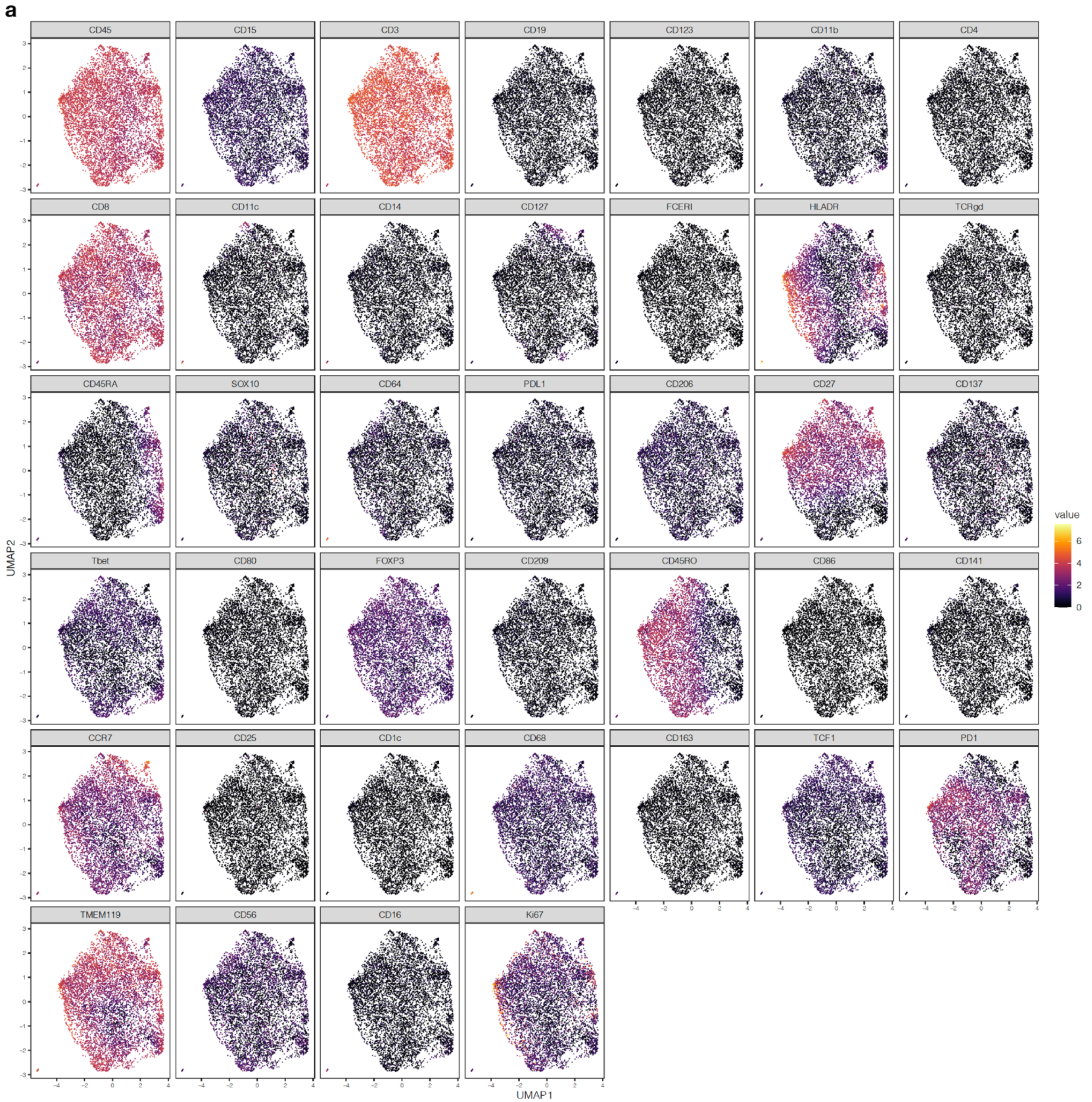

Supplementary Fig. 7. CyTOF CD8 T cell feature plots. Related to Fig. 1f, g. Legend shows asinh intensity. Source data are provided as a Source Data file.

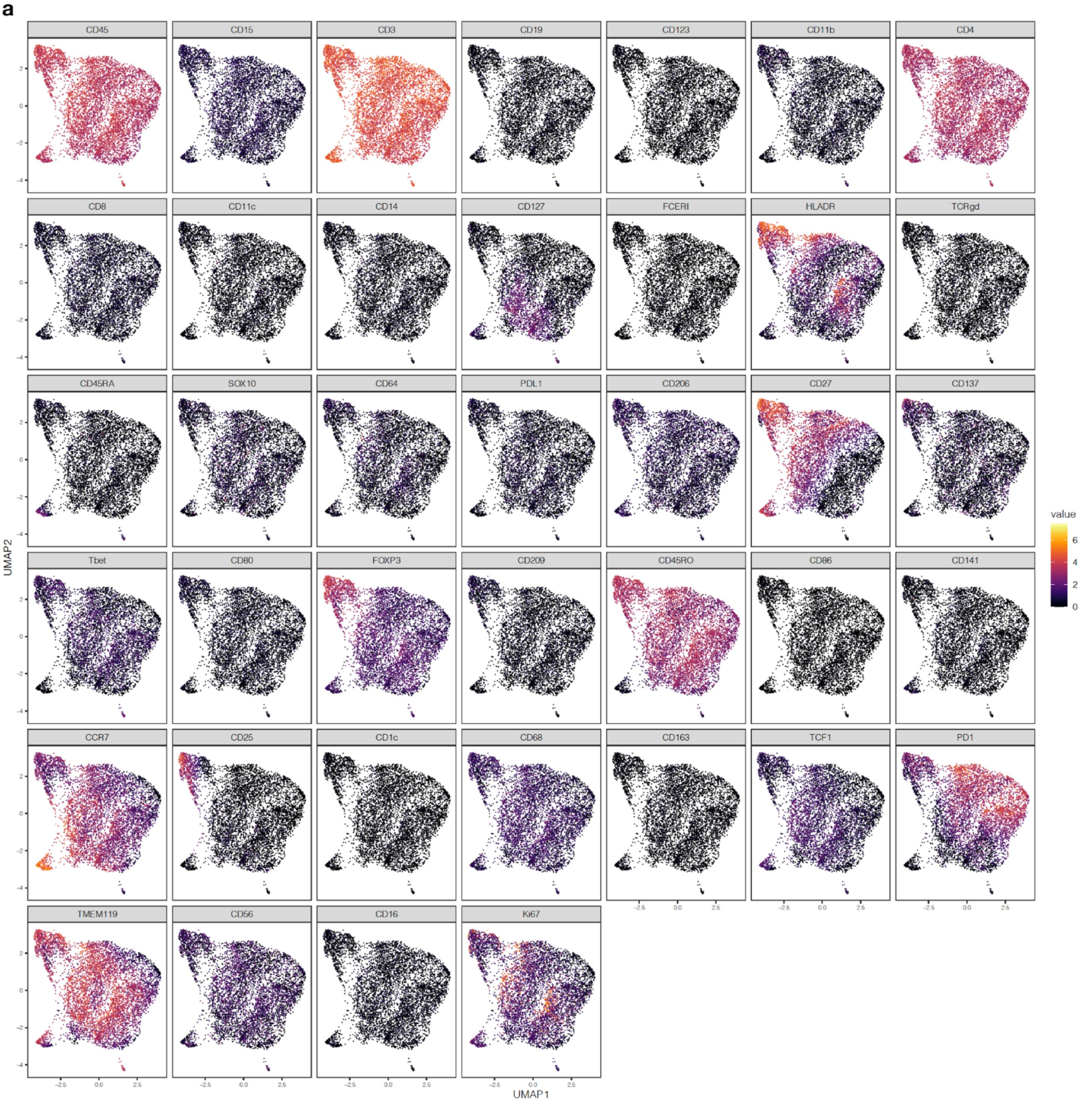

Supplementary Fig. 8. CyTOF feature plots used to define CD4 T cells. Related to Fig. 1f, g. No significant differences in CD4 T cell enrichment were identified between NCS and IES. Legend shows asinh intensity. Source data are provided as a Source Data file.

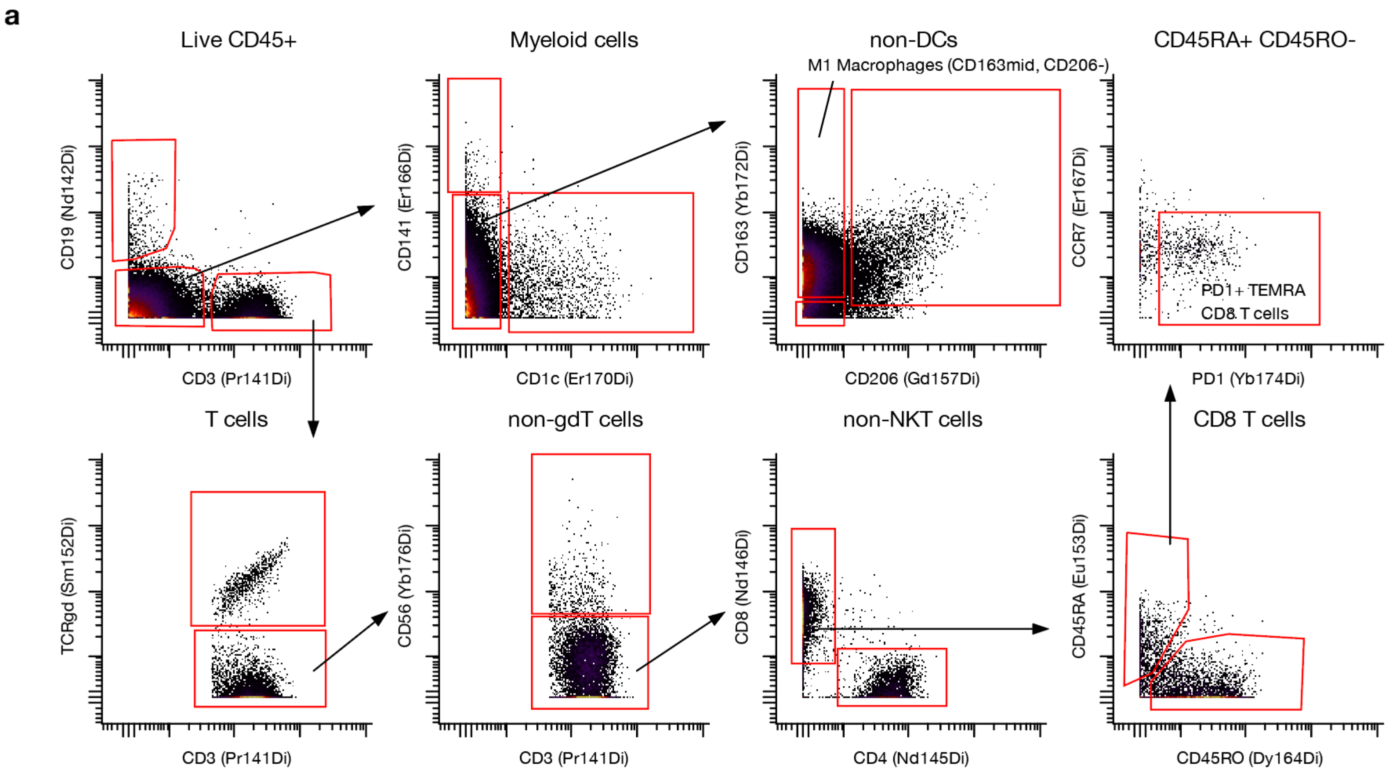

Supplementary Fig. 9. CyTOF gating workflow used to define schwannoma immune cell populations such as myeloid cells and CD8 T cells. Related to Fig. 1f, g.

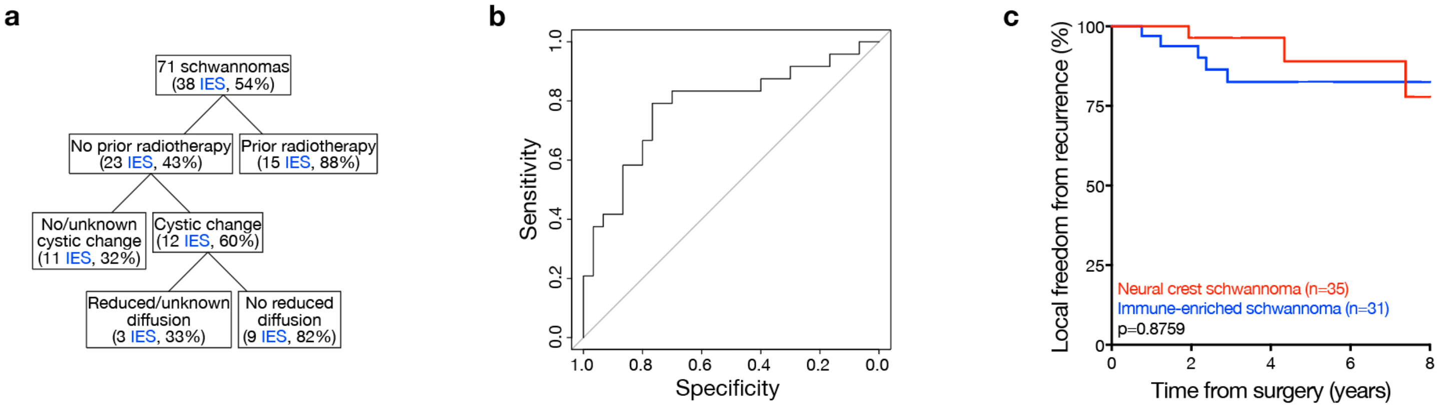

Supplementary Fig. 10. Analysis of clinical features distinguishing schwannoma molecular groups. a, Recursive partitioning analysis of clinical and magnetic resonance imaging features in patients with IES versus NCS (n=71). **b**, Receiver operating characteristic curve for logistic regression modeling to predict schwannoma molecular group based on non-invasive clinical and magnetic resonance imaging features (area under the curve 0.79). c, Kaplan-Meier curves for local freedom from recurrence for NCS versus IES demonstrating no significant difference in tumor control between molecular groups (log-rank test). Source data are provided as a Source Data file.

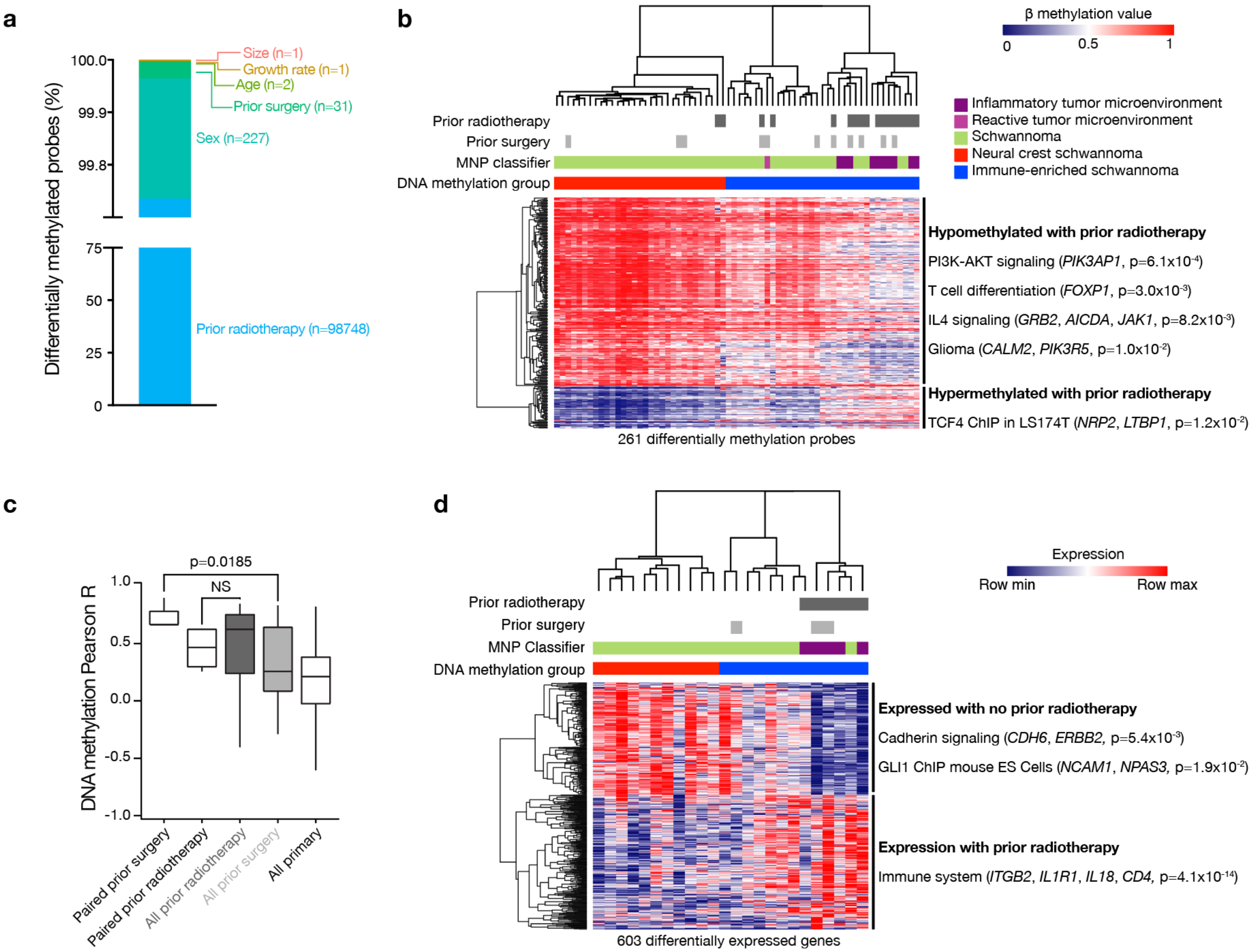

Supplementary Fig. 11. Radiotherapy is associated with epigenetic reprogramming of neural crest to immune-enriched schwannoma. a, Differentially methylated DNA probes based on clinical characteristics of schwannoma patients (n=66, FDR<0.05). **b**, Hierarchical clustering of differentially methylated DNA probes from schwannomas (n=66) with versus without prior radiotherapy (FDR<0.01). Significant gene ontology terms corresponding to hypomethylated probes, clinical metadata, and the molecular neuropathology (MNP) DNA methylation classification of central nervous system tumors[2] are shown. Hypermethylated genes associated with oncogenic Hedgehog signaling include *IL31RA*[21,22], *TRPM8*[21,23,24] *KCNH5*[21,24–26], and *FBLN2*[21–23,27,28]. **c**, Distributions of pairwise Pearson correlation coefficients for patient-matched pairs of primary and recurrent schwannomas (n=13). P-values determined using two-sided Kolmogorov-Smirnov test. Boxplots show 1st quartile, median, and 3rd quartile. Whiskers represent 1.5 inter-quartile range. **d**, Hierarchical clustering of differentially expressed genes from RNA sequencing of schwannomas with versus without prior radiotherapy (n=24). GLI1 transcriptional targets include *NCAM1* and *NPAS3*[28,29]. Significant gene ontology terms ± prior radiotherapy and meta-data are shown as in. **b**. Source data are provided as a Source Data file.

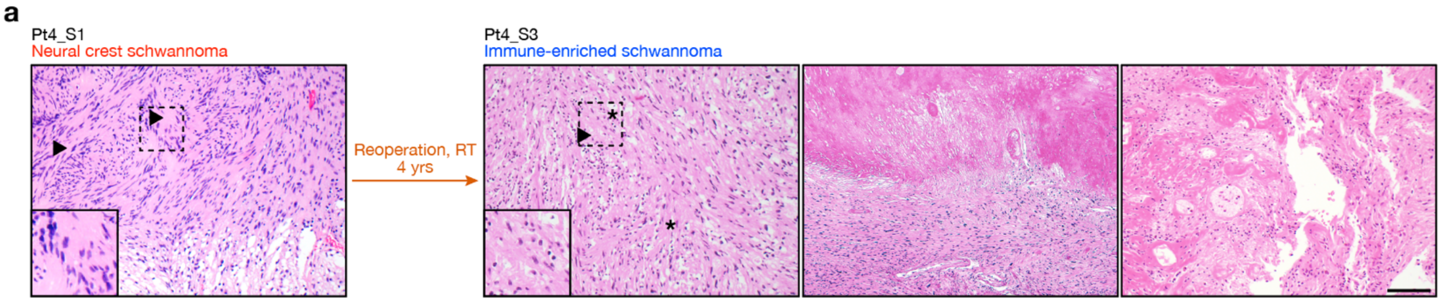

Supplementary Fig. 12. Radiotherapy durably recruits immune cells to the schwannoma microenvironment. a, H&E-stained sections of a patient-matched primary NCS and recurrent IES, showing typical histology with biphasic histology and Verocay bodies (left arrows) that was replaced by abundant lymphocytes (right arrows) with foamy and hemosiderin-filled macrophages (asterisks), coagulative necrosis (second from right), and hyalinized vessels (far right). RT, radiotherapy. Scale bar, 100μm. Similar results were obtained for a separate paired sampled in Fig. 2b.

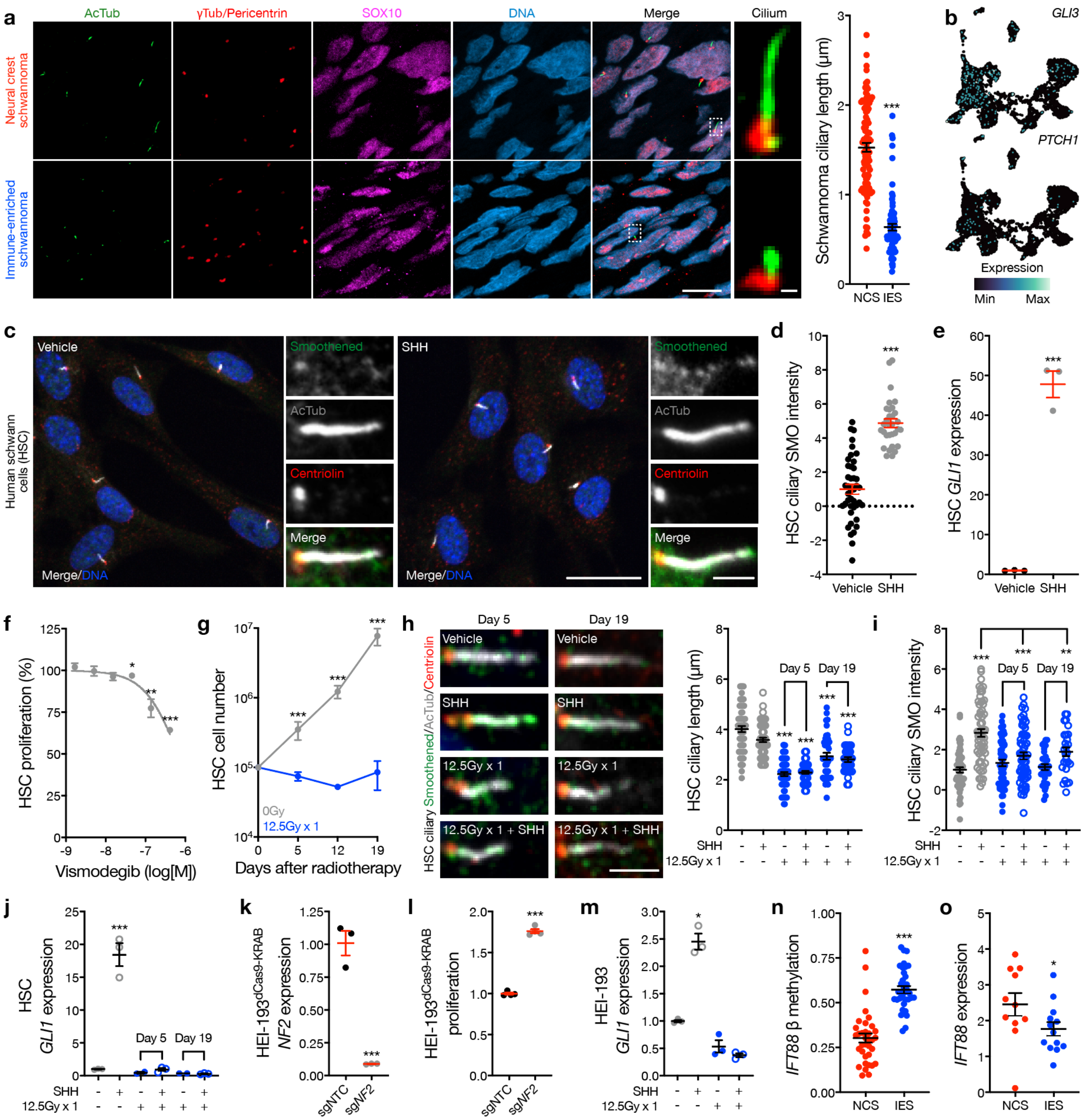

Supplementary Fig. 13. Radiotherapy inhibits Schwann and schwannoma cell Hedgehog signaling. a, Quantitative immunofluorescence microscopy for the ciliary axoneme marker acetylated tubulin (AcTub), the ciliary base markers gamma tubulin (γtub) and pericentrin (red), and the schwannoma cell marker SOX10 (purple) in 174 cilia from 6 schwannomas. Scale bars, 10μm and 1μm. ***p=5.06x10^{-29} **b**, Feature plot of integrated UMAP from harmonized schwannoma single-nuclei and single-cell RNA sequencing (Fig. 1b) showing expression of the Hedgehog target genes *GLI3* or *PTCH1* in schwannoma cells. **c** and **d**, Quantitative immunofluorescence microscopy for AcTub, the ciliary base marker Centriolin, and the Hedgehog pathway activator Smoothened in immortalized human Schwann cells (HSCs) treated with recombinant sonic Hedgehog (SHH) or vehicle control. Scale bars, 20μm and 2μm. n=42 vehicle, n=31 SHH cilia. ***p=1.04x10^{-14} **e**, QPCR for the Hedgehog target gene *GLI1* in HSC cultures treated with SHH or vehicle control. n=3 biological replicates. ***p=1.50x10^{-4} **f**, Quantification of HSC proliferation after treatment with the Smoothened antagonist vismodegib for 72h. Of note, vismodegib does not reduce ciliary length[30]. n=4 biological replicates. *p≤0.05, **p≤0.01,

***p≤0.0001. **g**, HSC proliferation after treatment with radiotherapy compared to control. n=3 biological replicates. ***day 5 p=0.010, day 12 p=0.0014, day 19 p=0.0035. **h** and **i**, Quantitative immunofluorescence imaging of primary cilia in HSC cultures 5 days or 19 days after treatment with radiotherapy or control, and SHH or vehicle control. n=27-60 cilia quantified. ***ciliary length, day 5, vehicle + 12.5Gy x1 $p=5.75x10^{-22}$; ciliary length, day 5, SHH + 12.5Gy x1 $p=7.98x10^{-23}$; ciliary length, day 19, vehicle + 12.5Gy x1 $p=8.44x10^{-8}$; ciliary length, day 19, SHH + 12.5Gy x1 $p=4.76x10^{-7}$; SMO intensity, SHH $p=3.01x10^{-12}$; SMO intensity, day 5, SHH + 12.5Gy x1 $p=2.74x10^{-5}$; SMO intensity, day 19, SHH + 12.5Gy x1 p=0.0053. **j**, GLI1 QPCR in HSC cultures 5 days or 19 days after treatment with radiotherapy or control, and SHH or vehicle control. n=2-3 biological replicates. ***$p=5.92x10^{-4}$. **k**, QPCR validation of *NF2* CRISPRi suppression in HEI-193 schwannoma cells compared to non-targeting control sgRNAs (sgNTC). n=3 biological replicates. ***$p=6.02x10^{-4}$. **l**, Quantification of HEI-193 cell proliferation following CRISPRi suppression of *NF2* compared to sgNTC. n=4 biological replicates. ***$p=1.94x10^{-7}$. **m**, GLI1 QPCR in HEI-193 cells 5 days after treatment with radiotherapy or control plus SHH or vehicle control. n=3 biological replicates. ***$p=6.34x10^{-4}$. **n**, DNA methylation β values for the ciliary axoneme gene *IFT88* in NCS compared to IES. n=35 independent tumors per type. ***$p=6.38x10^{-12}$. IFT88 is associated with the centrosome throughout the cell cycle and control cells proliferation by regulating the G1-S transition. As such, downregulation of *IFT88* in cultured human and mouse cells induces mitotic defects *in vitro*[31,32]. **o**, RNA sequencing transcripts per million expression of *IFT88* in NCS compared to IES. n=11 NCS, n=13 IES independent tumors. *p<0.05. Lines represent means and error bars represent standard error of means (Two-sided Student's t tests used). Biological replicates are shown by individual data points. Source data are provided as a Source Data file.

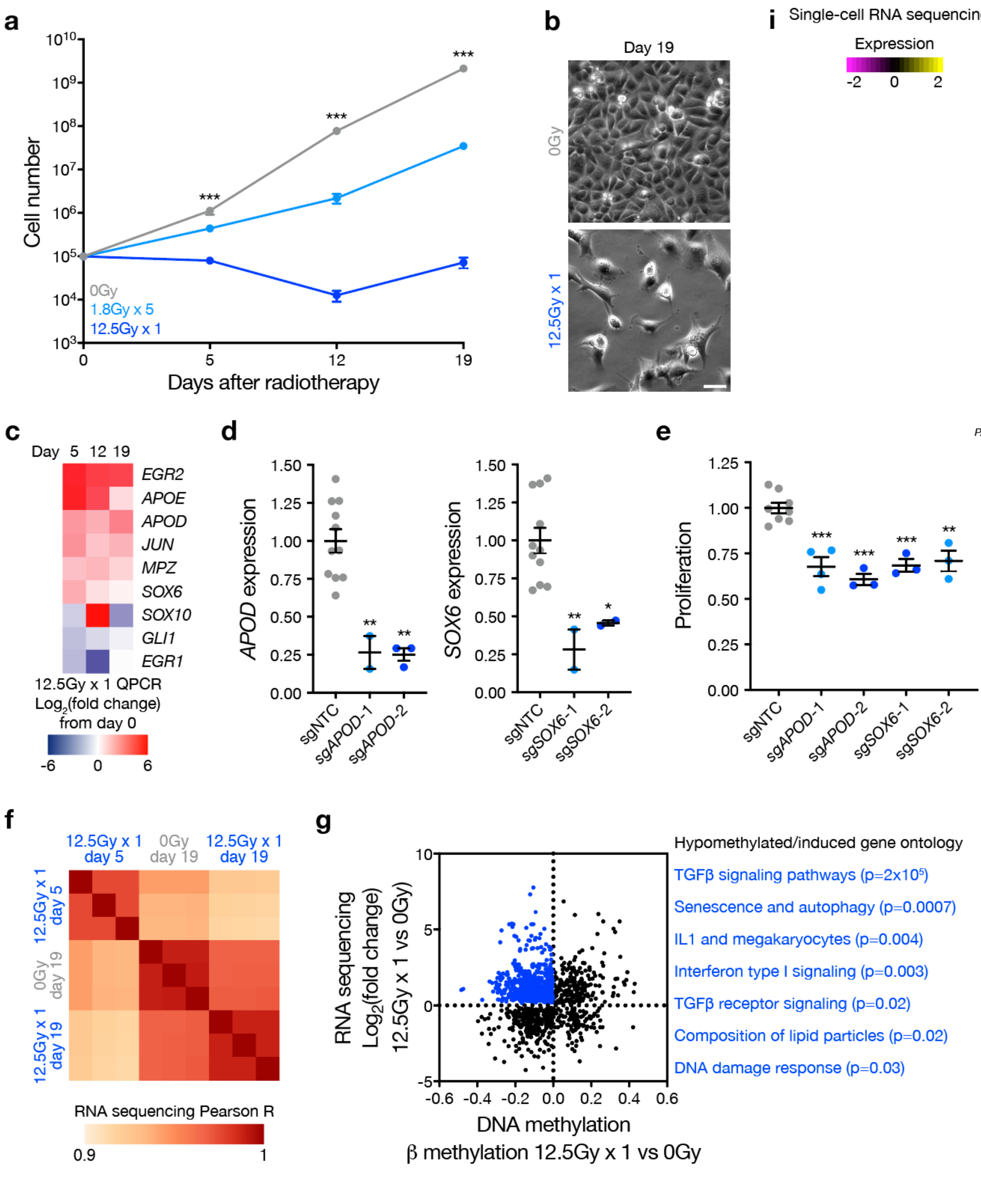

h

Conditions	C0	C1	C2	C3	C4	C5	C6	C7	C8	C9	C10	C11	C12	C13	C14	Total
0Gy	1266	696	306	678	590	225	352	373	111	48	22	9	48	46	0	4770
0Gy	1122	633	307	619	590	215	336	431	93	59	7	3	36	33	0	4484
0Gy	1302	708	403	699	740	219	373	506	106	57	12	14	32	61	0	5232
1.8Gy x5	618	517	347	416	516	698	304	283	98	84	18	14	32	69	0	4014
1.8Gy x5	358	402	337	330	505	669	238	201	122	92	13	124	24	43	0	3458
1.8Gy x5	796	467	456	492	537	814	325	316	107	92	51	282	32	60	0	4827
12.5Gy x1	1266	764	728	280	206	121	198	26	265	78	30	11	82	25	29	4109
12.5Gy x1	1122	932	848	333	257	94	231	31	372	45	27	5	94	37	0	4428
12.5Gy x1	1302	629	771	263	163	110	184	18	208	149	335	7	76	32	0	4247
**Total**	9152	5748	4503	4110	4104	3165	2541	2185	1482	704	515	469	456	406	29	39569

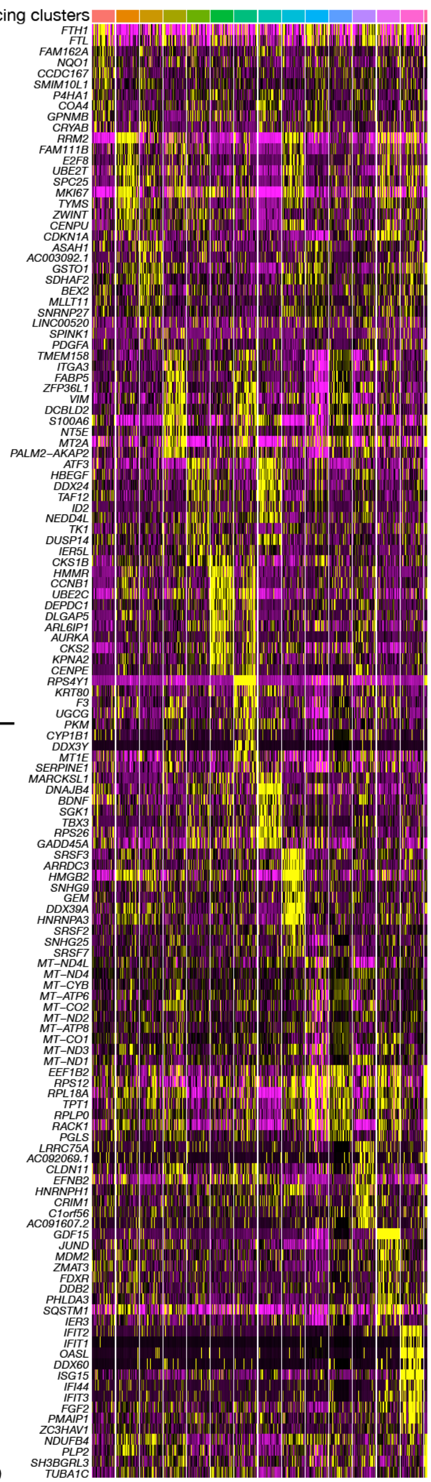

Supplementary Fig. 14. Radiotherapy epigenetically reprograms schwannoma cells to multiple cellular states, expressing immune and inflammatory genes that regulate schwannoma cell growth. a, Quantification of HEI-193 schwannoma cell proliferation over time after treatment with 0Gy, 1.8Gy x 5, or 12.5Gy x1 (n=3 independent cultures per condition, ***p=0.00084 day 5, p=0.00038 day 12, p=0.00041 day 19). **b**, Light microscopy of HEI-193 cells treated with either 0Gy or 12.5Gy x1 of radiotherapy. Irradiated cells demonstrate evidence of senescence-associated, fried-egg morphology. Scale bar, 10µm. Similar micrographs were observed in n=3 independent cultures for each condition. **c**, Heatmap of $\log_2$ fold changes of QPCR gene expression between day 0 and day 5, 12 or 19 following 12.5Gy x1 of radiotherapy treatment of HEI-193 cells. Heatmap values represent averages of 3 QPCR replicates per condition. **d**, CRISPRi suppression *APOD* or *SOX6* (n=2-3 biological replicates per sgRNA, **p=0.00041 sg*APOD*-1, p=0.00035 sg*APOD*-2, p=0.00092 sg*SOX6*-1, *p=0.022 sg*SOX6*-2) in HEI-193 cells compared to non-targeted control sgRNAs (sgNTC, n=11 biological replicates). **e**, Quantification of HEI-193 cell proliferation after CRISPRi repression of *APOD* or *SOX6* (n=3-4 biological replicates per sgRNA, ***p=0.00016 sg*APOD*-1, p=0.000036 sg*APOD*-2, p= 0.00021 sg*SOX6*-

1, **p= 0.00076 sg*SOX6*-2) using 2 independent sgRNAs per gene as in **d**, compared to sgNTC (n=8 biological replicates) . Lines represent means and error bars represent standard error of means (Two-sided Student's t test used). **f**, Heatmap of pairwise Pearson correlation coefficients between RNA sequencing of triplicate HEI-193 cultures after treatment with either 0Gy or 12.5Gy x 1 radiotherapy, 5- or 19-days following radiation treatment. **g**, Comparison of RNA sequencing and DNA methylation profiling of HEI-193 cells 19 days after 12.5Gy x 1 of radiotherapy compared to 0Gy control treatment. Significant gene ontology terms for enriched and hypomethylated genes are shown (blue, n=3 replicate cultures per condition, two-sided Fisher's exact test). **h**, Distribution of HEI-193 single-cell RNA sequencing transcriptomes (Fig. 2c). **i**, Expression heatmap of schwannoma cell state marker genes from single-cell RNA sequencing of triplicate HEI-193 cultures following 0Gy, 1.8Gy x 5, or 12.5Gy x 1 radiotherapy. Columns represent transcriptomes from single cells downsampled to 200 cells per cluster. Source data are provided as a Source Data file.

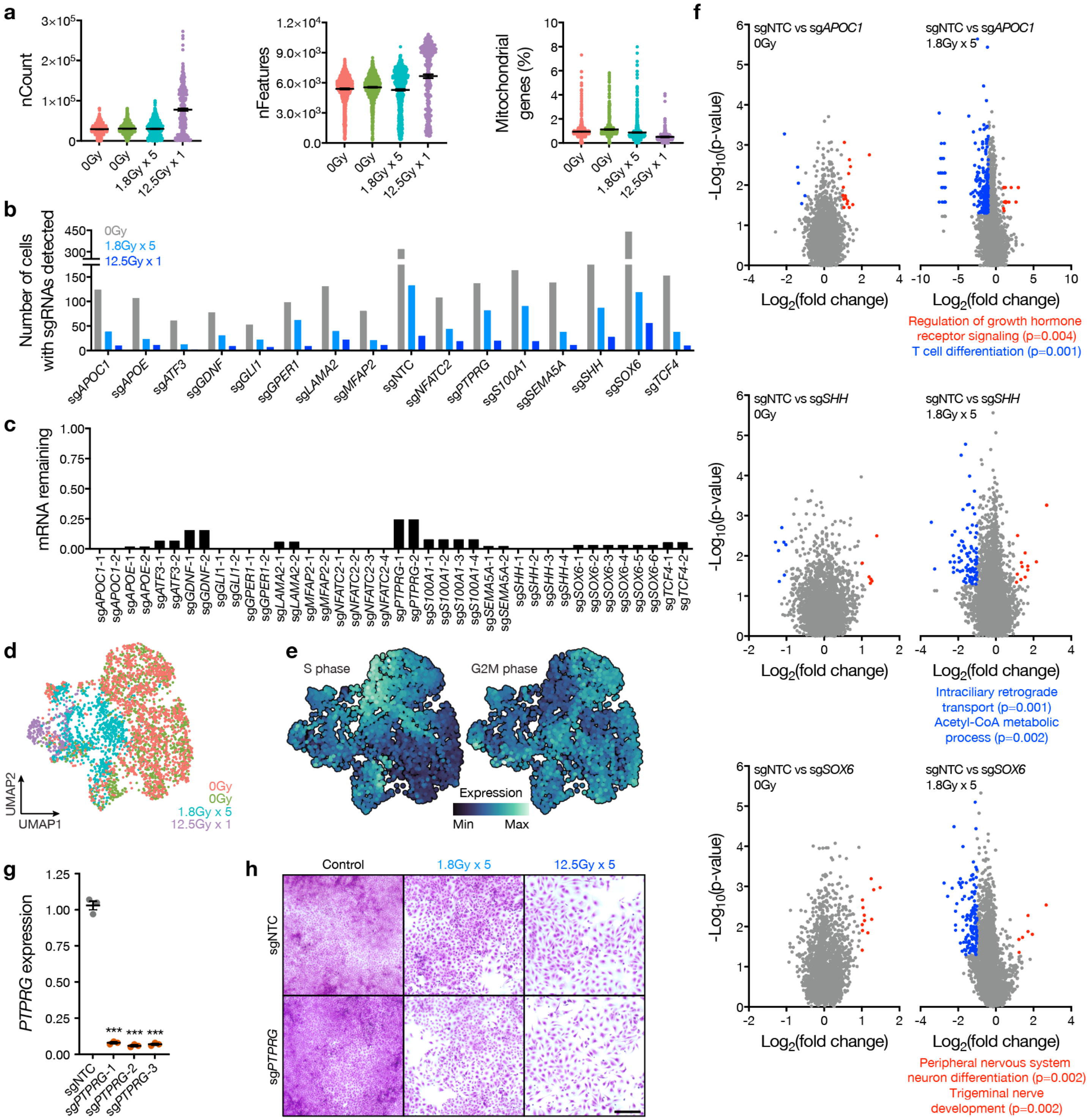

Supplementary Fig. 15. Perturb-seq of schwannoma marker genes reveals regulators of cell states. a, Distributions of UMI counts (left), number of unique gene expression features (middle), and percentage of UMIs mapping to mitochondria gene (right) from Perturb-seq in HEI-193 cells. **b**, sgRNA coverage for each targeted gene in 0Gy, 1.8Gy x 5, or 12.5Gy x 1 radiotherapy conditions. n=2-6 sgRNAs per gene targeted. **c**, Mean knockdown of targeted genes in cells pseudobulked by each sgRNA. Only sgRNAs with confirmed repression of greater than 75% were retained for analysis. **d**, UMAP of 3546 HEI-193 cells passing quality thresholds for Perturb-seq. **e**, Feature plot of S phase or G2M phase genes in Perturb-seq UMAP from **d**. **f**, Volcano plots for differential gene expression analysis using MAST for *APOC1, SHH,* or *SOX6* perturbations compared to sgNTC in 0Gy (left) versus 1.8Gy x 5 conditions (right). Significant positive (red) or negative (blue) gene expression changes are colored ($p<0.05$, $|\log_2(\text{fold change})|>1$), corresponding to gene ontology terms (Two-sided Fisher's exact test). **g**, CRISPRi suppression of *PTPRG* in HEI-193 cells compared to non-targeted control sgRNAs (sgNTC) (n=3 independent cultures, ***$p=6.83\times10^{-6}$ sg*PTPRG*-1, $p=6.29\times10^{-6}$ sg*PTPRG*-2, $p=6.55\times10^{-6}$ sg*PTPRG*-3). **h**, Representative crystal violet staining of HEI-193 cells following CRISPRi suppression of *PTPRG*

compared to sgNTC after treatment with 0Gy, 1.8Gy x 5, or 12.5Gy x 1 of radiotherapy. Irradiated cells demonstrate evidence of senescence-associated, fried-egg morphology. Similar micrographs were obtained in n=3 independent cultures for each condition. Scale bar, 100µm. Lines represent means and error bars represent standard error of means (Two-sided Student's t tests). Source data are provided as a Source Data file.

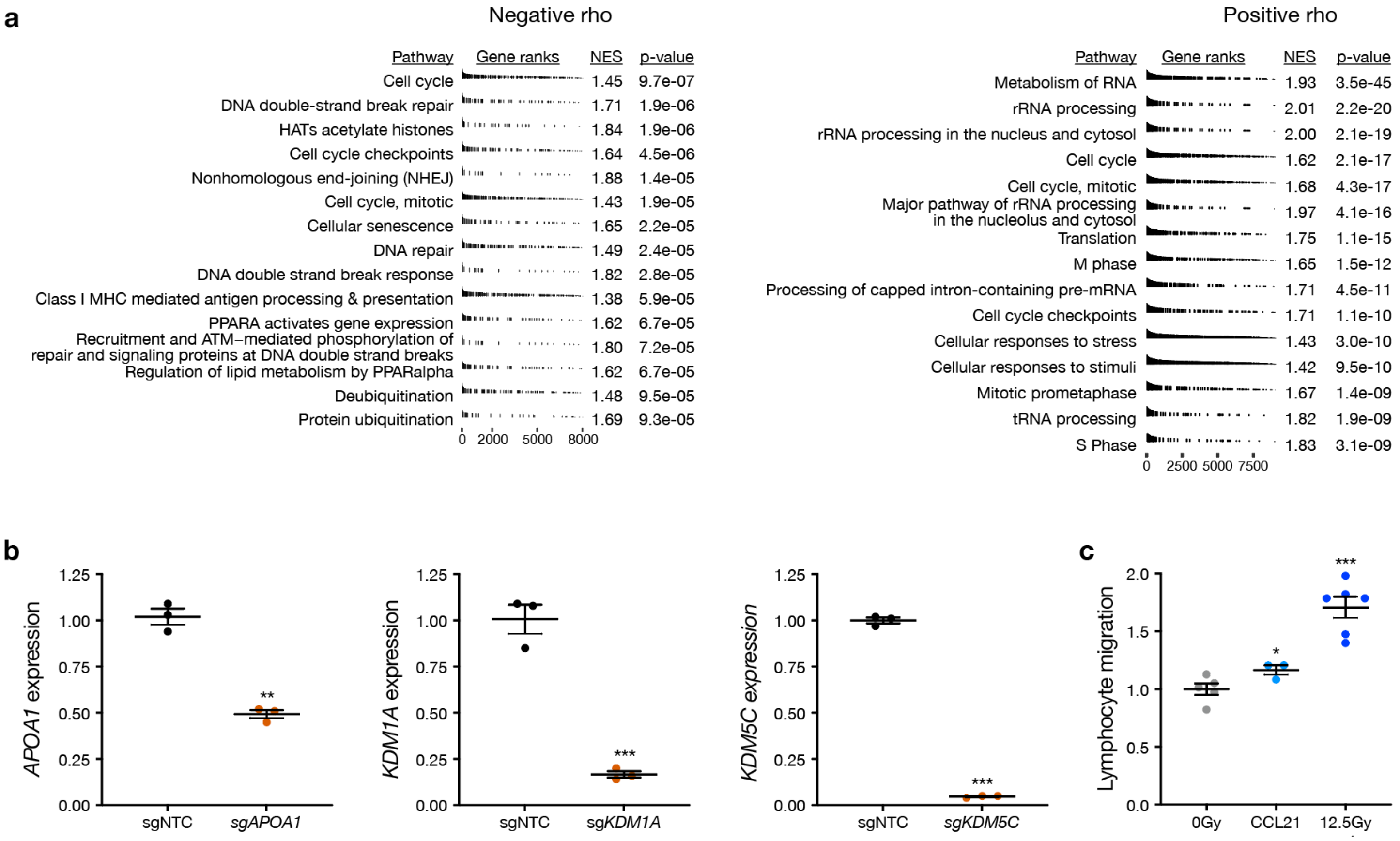

Supplementary Fig. 16. Genome-wide CRISPRi screens reveal regulators of schwannoma cell radiotherapy responses. a, Gene set enrichment analysis of ranked gene targets exhibiting negative rho (left, radiotherapy sensitivity) or positive rho (right, radiotherapy resistance) following CRISPRi gene suppression. Gene ontology terms derived from Reactome. NES, normalized enrichment score. **b**, CRISPRi suppression of *APOA1*, *KDM1A*, or *KDM5C* in HEI-193 cells (n=3 independent cultures, **p=0.00042 *sgAPOA1,* ***p= 0.00047 sg*KDM1A*, p=4.33x10^{-7} sg*KDM5C*). **c**, Transwell primary human peripheral blood lymphocyte migration assays using conditioned media from HEI-193 cells ± radiotherapy or with recombinant CCL21 as a chemoattractant (*p=0.033, ***p=0.000125). Lines represent means and error bars represent standard error of means. (Two-sided Student's t tests used). Source data are provided as a Source Data file.

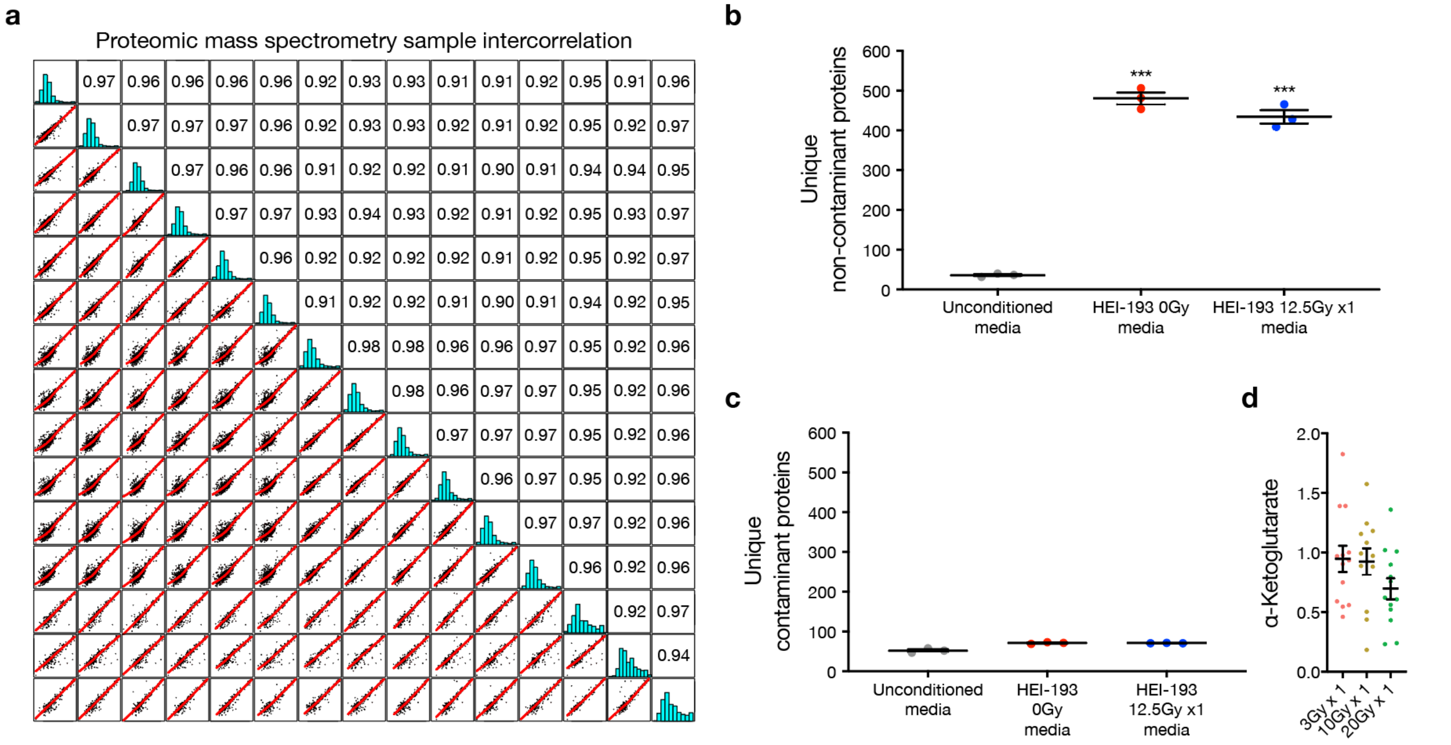

Supplementary Fig. 17. Proteomic and targeted metabolic mass spectrometry of schwannoma cells. a, Proteomic mass spectrometry Pearson correlation matrix from HSC and HEI-193 media demonstrating high correlation across samples. **b** and **c**, Unique non-contaminant or contaminant protein counts from HEI-193 media mass spectrometry (n=3 independent cultures, Two-sided Student's t tests, ***p=8.17×10^{-6} 0Gy, p=1.94×10^{-5} 12.5Gy). **d**, Metabolite mass spectrometry of primary patient-derived human schwannoma cells (n=7 patient samples) after treatment with 3Gy x 1, 10Gy x 1, or 20Gy x 1 of radiotherapy validated suppression of α-Ketoglutarate with ionizing radiation (Fig. 3i). Fold changes normalized to 0Gy treatment for each primary cell culture (ANOVA, $p \leq 0.05$). Lines represent means and error bars represent standard error of means. Source data are provided as a Source Data file.

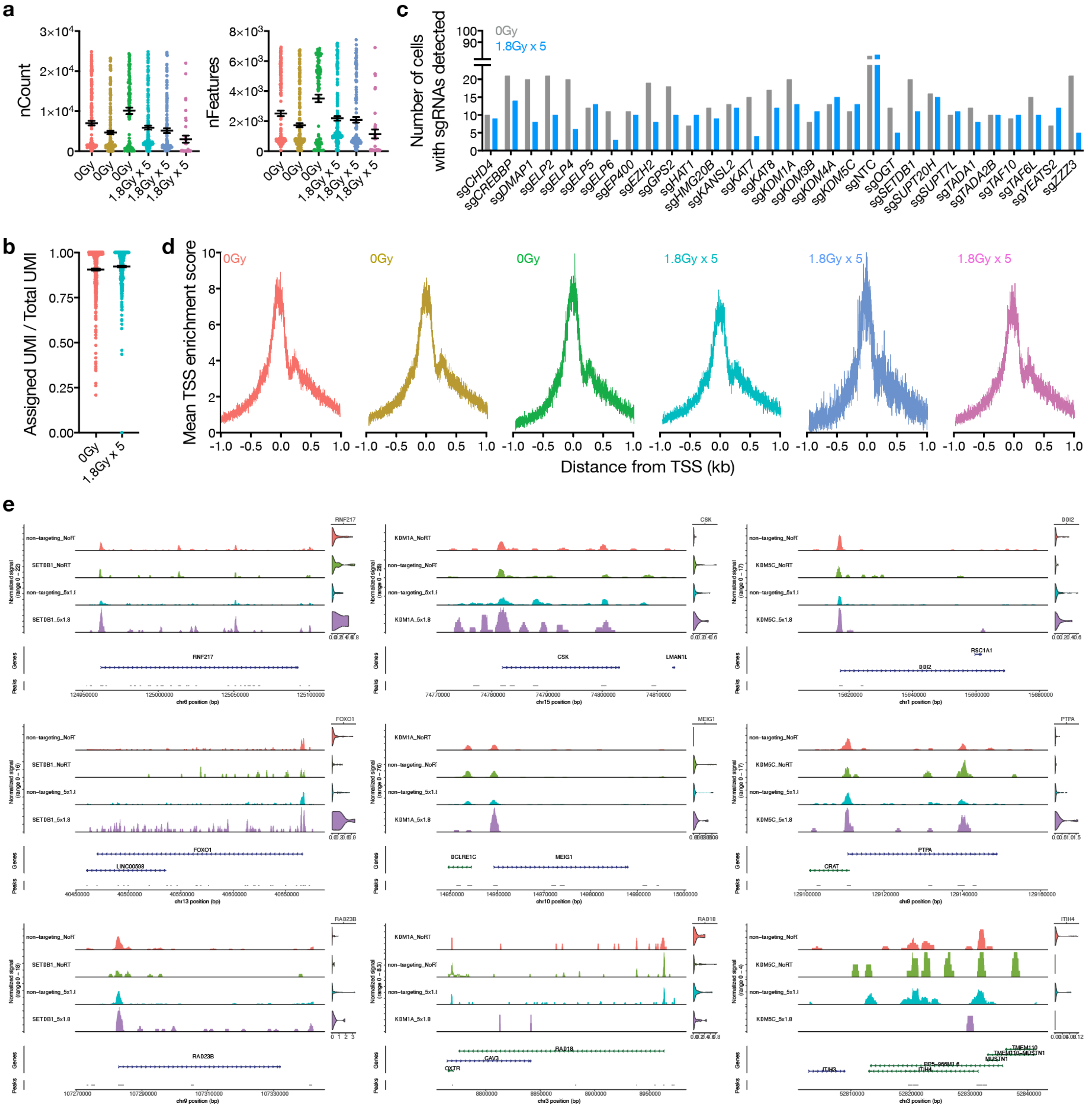

Supplementary Fig. 18. snARC-seq quality metrics for simultaneous profiling of chromatin accessibility and gene expression in the context of therapeutic and CRISPRi perturbations. a, Distributions of UMI count (left) and number of unique gene expression features (right) from snARC-seq in HEI-193 cells (n=489 cells in 0Gy, n=366 cells in 1.8Gy x 5, lines represent means and error bars represent standard error of means). **b**, Distributions of UMIs mapping to sgRNAs as a proportion of all sgRNA UMI counts in individual HEI-193 cells. Lines represent means and error bars represent standard error of means. **c**, sgRNA coverage for each targeted gene in 0Gy or 1.8Gy x 5 radiotherapy conditions. **d**, Transcription start site (TSS) enrichment profile plots for snARC-seq ATAC chromatin accessibility profiles, demonstrating characteristic nucleosome-free regions relative to the TSS in each condition. **e**, Pseudobulked ATAC chromatin accessibility profiles coupled with single-nuclei RNA expression distributions at example genomic loci of genes that were activated with *SETDB1* perturbation (left), activated or repressed with *KDM1A* perturbation (middle), or activated or repressed with *KDM5C* perturbation (right), in the presence or absence of radiotherapy (1.8Gy x 5). Source data are provided as a Source Data file.

Supplementary References

1. Agnihotri, S. *et al.* The genomic landscape of schwannoma. *Nature Genetics* 48, 1339–1348 (2016).
2. Capper, D. *et al.* DNA methylation-based classification of central nervous system tumours. *Nature* 555, 469–474 (2018).
3. Ohta, S. *et al.* Macrophage migration inhibitory factor (MIF) promotes cell survival and proliferation of neural stem/progenitor cells. *J Cell Sci* 125, 3210–3220 (2012).
4. Zhu, Z. *et al.* Macrophage Migration Inhibitory Factor Promotes Chemotaxis of Astrocytes through Regulation of Cholesterol 25-Hydroxylase Following Rat Spinal Cord Injury. *Neuroscience* 408, 349–360 (2019).
5. Mi, S. *et al.* LINGO-1 negatively regulates myelination by oligodendrocytes. *Nat Neurosci* 8, 745–751 (2005).
6. Mi, S. *et al.* LINGO-1 antagonist promotes spinal cord remyelination and axonal integrity in MOG-induced experimental autoimmune encephalomyelitis. *Nat Med* 13, 1228–1233 (2007).
7. Zhu, D., Hunter, S. B., Vertino, P. M. & Meir, E. G. V. Overexpression of MBD2 in Glioblastoma Maintains Epigenetic Silencing and Inhibits the Antiangiogenic Function of the Tumor Suppressor Gene BAI1. *Cancer Res* 71, 5859–5870 (2011).
8. Cayé-Thomasen, P. *et al.* VEGF and VEGF Receptor-1 Concentration in Vestibular Schwannoma Homogenates Correlates to Tumor Growth Rate. *Otol Neurotol* 26, 98–101 (2005).
9. Aust, G., Zhu, D., Meir, E. G. V. & Xu, L. Adhesion G Protein-coupled Receptors, Molecular, Physiological and Pharmacological Principles in Health and Disease. *Handb Exp Pharmacol* 234, 369–396 (2016).
10. Wong, H.-K. *et al.* Merlin/NF2 Regulates Angiogenesis in Schwannomas through a Rac1/Semaphorin 3F-Dependent Mechanism. *Neoplasia* 14, 84–94 (2012).
11. Parrinello, S. *et al.* NF1 loss disrupts Schwann cell–axonal interactions: a novel role for semaphorin 4F. *Gene Dev* 22, 3335–3348 (2008).
12. Schulz, A. *et al.* The importance of nerve microenvironment for schwannoma development. *Acta Neuropathol* 132, 289–307 (2016).
13. Glerup, S. *et al.* SorCS2 Regulates Dopaminergic Wiring and Is Processed into an Apoptotic Two-Chain Receptor in Peripheral Glia. *Neuron* 82, 1074–1087 (2014).
14. Taylor, A. M. *et al.* Axonal mRNA in Uninjured and Regenerating Cortical Mammalian Axons. *J Neurosci* 29, 4697–4707 (2009).
15. Han, B. H. *et al.* Clusterin contributes to caspase-3–independent brain injury following neonatal hypoxia-ischemia. *Nat Med* 7, 338–343 (2001).
16. Gupta, S. D., Lipponen, A., Paldanius, K. M. A., Puhakka, N. & Pitkänen, A. Dynamics of clusterin protein expression in the brain and plasma following experimental traumatic brain injury. *Sci Rep-uk* 9, 20208 (2019).
17. White, T. E. *et al.* Gene expression patterns following unilateral traumatic brain injury reveals a local pro-inflammatory and remote anti-inflammatory response. *Bmc Genomics* 14, 282 (2013).
18. Yuzwa, S. A. *et al.* Developmental Emergence of Adult Neural Stem Cells as Revealed by Single-Cell Transcriptional Profiling. *Cell Reports* 21, 3970–3986 (2017).
19. Hung, H. A., Sun, G., Keles, S. & Svaren, J. Dynamic regulation of Schwann cell enhancers after peripheral nerve injury. *Journal of Biological Chemistry* 290, 6937–6950 (2015).
20. Liberzon, A. *et al.* The Molecular Signatures Database Hallmark Gene Set Collection. *Cell Syst* 1, 417–425 (2015).
21. Raleigh, D. R. *et al.* Hedgehog signaling drives medulloblastoma growth via CDK6. *J Clin Invest* 128, 120–124 (2017).
22. Kool, M. *et al.* Genome Sequencing of SHH Medulloblastoma Predicts Genotype-Related Response to Smoothened Inhibition. *Cancer Cell* 25, 393–405 (2014).
23. Robinson, G. *et al.* Novel mutations target distinct subgroups of medulloblastoma. *Nature* 488, 43–48 (2012).
24. Northcott, P. A. *et al.* Medulloblastomics: the end of the beginning. *Nat Rev Cancer* 12, 818–834 (2012).
25. Pugh, T. J. *et al.* Medulloblastoma exome sequencing uncovers subtype-specific somatic mutations. *Nature* 488, 106–110 (2012).
26. Hovestadt, V. *et al.* Decoding the regulatory landscape of medulloblastoma using DNA methylation sequencing. *Nature* 510, 537–541 (2014).
27. Thompson, M. C. *et al.* Genomics Identifies Medulloblastoma Subgroups That Are Enriched for Specific Genetic Alterations. *J Clin Oncol* 24, 1924–1931 (2006).
28. Vokes, S. A. *et al.* Genomic characterization of Gli-activator targets in sonic hedgehog-mediated neural patterning. *Development* 134, 1977–1989 (2007).
29. Lachmann, A. *et al.* ChEA: transcription factor regulation inferred from integrating genome-wide ChIP-X experiments. *Bioinformatics* 26, 2438–2444 (2010).

30. Maurya, D. K., Bohm, S. & Alenius, M. Hedgehog signaling regulates ciliary localization of mouse odorant receptors. *Proc National Acad Sci* 114, E9386–E9394 (2017).
31. Robert, A. *et al.* The intraflagellar transport component IFT88/polaris is a centrosomal protein regulating G1-S transition in non-ciliated cells. *J Cell Sci* 120, 628–637 (2007).
32. Delaval, B., Bright, A., Lawson, N. D. & Doxsey, S. The cilia protein IFT88 is required for spindle orientation in mitosis. *Nat Cell Biol* 13, 461–468 (2011).
