# Supplementary material for: Epigenetic reprogramming shapes the cellular landscape of schwannoma
Source: Nat Commun. 2024 Jan 12;15:476. doi: 10.1038/s41467-023-40408-5 (PMC10786948; doi:10.1038/s41467-023-40408-5)
Supplement: Supplementary file 3 — Description of Additional Supplementary Files [file 41467_2023_40408_MOESM3_ESM.pdf]

Description of Additional Supplementary Files

Supplementary Data 1. Patient and vestibular schwannoma characteristics.

Supplementary Data 2. Gene ontology terms from schwannoma methylation groups.

Supplementary Data 3. Differentially expressed genes from RNA-sequencing of immune-enriched (positive) vs neural crest (negative) schwannomas.

Supplementary Data 4. Top marker genes from integrated schwannoma single cell RNA- and single nuclei RNA-sequencing clusters.

Supplementary Data 5. Antibodies and concentrates used for mass cytometry time-of-flight-analysis.

Supplementary Data 6. Differentially expressed genes from RNA-seq of schwannomas with prior radiotherapy (positive) vs no radiotherapy (negative).

Supplementary Data 7. Top marker genes from single cell RNA-sequencing of irradiated HEI-193 cells.

Supplementary Data 8. Perturb-seq sgRNAs and gene targets.

Supplementary Data 9. Differential gene expression analysis from Perturb-seq of schwannoma marker genes (positive) vs control sgRNA (negative).

Supplementary Data 10. Genome wide CRISPR interefence screen in HEI-193 cells +/- radiotherapy.

Supplementary Data 11. QPCR primer and sgRNA protospacer sequences.
