# Supplementary material for: Epigenetic reprogramming shapes the cellular landscape of schwannoma
Source: Nat Commun. 2024 Jan 12;15:476. doi: 10.1038/s41467-023-40408-5 (PMC10786948; doi:10.1038/s41467-023-40408-5)
Supplement: Supplementary file 5 — Reporting Summary [file 41467_2023_40408_MOESM5_ESM.pdf]

nature portfolio

Corresponding author(s): David Raleigh

Last updated by author(s): May 1, 2023

Reporting Summary

Nature Portfolio wishes to improve the reproducibility of the work that we publish. This form provides structure for consistency and transparency in reporting. For further information on Nature Portfolio policies, see our Editorial Policies and the Editorial Policy Checklist.

Statistics

For all statistical analyses, confirm that the following items are present in the figure legend, table legend, main text, or Methods section.

n/a	Confirmed	
☐	☒	The exact sample size (*n*) for each experimental group/condition, given as a discrete number and unit of measurement
☐	☒	A statement on whether measurements were taken from distinct samples or whether the same sample was measured repeatedly
☐	☒	The statistical test(s) used AND whether they are one- or two-sided<br>*Only common tests should be described solely by name; describe more complex techniques in the Methods section.*
☐	☒	A description of all covariates tested
☐	☒	A description of any assumptions or corrections, such as tests of normality and adjustment for multiple comparisons
☐	☒	A full description of the statistical parameters including central tendency (e.g. means) or other basic estimates (e.g. regression coefficient) AND variation (e.g. standard deviation) or associated estimates of uncertainty (e.g. confidence intervals)
☐	☒	For null hypothesis testing, the test statistic (e.g. *F*, *t*, *r*) with confidence intervals, effect sizes, degrees of freedom and *P* value noted<br>*Give P values as exact values whenever suitable.*
☒	☐	For Bayesian analysis, information on the choice of priors and Markov chain Monte Carlo settings
☐	☒	For hierarchical and complex designs, identification of the appropriate level for tests and full reporting of outcomes
☐	☒	Estimates of effect sizes (e.g. Cohen's *d*, Pearson's *r*), indicating how they were calculated

Our web collection on statistics for biologists contains articles on many of the points above.

Software and code

Policy information about availability of computer code

Data collection	No software used for data collection.
Data analysis	DNA methylation profiling and analysis: minfi version 1.30 in R version 3.6.0, caret version 6.0.<br>CyTOF: Statistical Scaffold R v 0. 1 package (github.com/SpitzerLab/statisticalScaffold)<br>Proteomic mass spectrometry: Skyline 4.2.0.19072<br>Metabolomic mass spectrometry: TraceFinder 4.1<br>Single-cell/single-nuclei RNA sequencing and analysis: Cell Ranger version 1.3.1 (l0X Genomics), Seurat version 3.0<br>CRISPRi screen analysis: ScreenProcessing (https://github.com/mhorlbeck/ScreenProcessing)<br>Perturb-seq: Cell Ranger version 6.1.2 with sgRNA barcoding (l0X Genomics), Seurat 4.3.0 in R version 4.2.2<br>snARC-seq: Cell Ranger ARC version 2.0.1 (l0X Genomics), Signac vl.8.0, Geomux (https://github.com/noamteyssier/geomux); kallisto bustools (v0.24.1)<br>Custom code: https://github.com/liujohn/schwannoma

For manuscripts utilizing custom algorithms or software that are central to the research but not yet described in published literature, software must be made available to editors and reviewers. We strongly encourage code deposition in a community repository (e.g. GitHub). See the Nature Portfolio guidelines for submitting code & software for further information.

Data

Policy information about availability of data

All manuscripts must include a data availability statement. This statement should provide the following information, where applicable:

- Accession codes, unique identifiers, or web links for publicly available datasets
- A description of any restrictions on data availability
- For clinical datasets or third party data, please ensure that the statement adheres to our policy

DNA methylation array data are deposited on GEO under accession GSE222042 (https://www.ncbi.nlm.nih.gov/geo/query/acc.cgi?acc=GSE222042). The Hg19 human reference genome was used to analyzed DNA methylation arrays (https://bioconductor.org/packages/IlluminaHumanMethylationEPICanno.ilm10b2.hg19/). All sequencing raw data, including bulk RNA-seq, scRNA-seq, snRNA-seq, Perturb-seq, and snARC-seq using the illumina Hiseq 4000 and Novaseq 6000 sequencers, are deposited on SRA under accession PRJNA917076 (https://www.ncbi.nlm.nih.gov/bioproject/PRJNA917076/). Proteomic mass spectrometry data have been deposited on ProteomeXchange under accession PXD014798 (https://www.ebi.ac.uk/pride/archive/projects/PXD014798). Metabolomic mass spectrometry data have been deposited on MassIVE under accession MSV000091760 (ftp://massive.ucsd.edu/MSV000091760/). CyTOF raw data are deposited on (https://doi.org/10.17632/hjmvnf48gh.1). Source data are provided with this paper.

Research involving human participants, their data, or biological material

Policy information about studies with human participants or human data. See also policy information about sex, gender (identity/presentation), and sexual orientation and race, ethnicity and racism.

Reporting on sex and gender	Sex is indicated for all clinical tumor samples. 26 out of 66 patient specimens in the discovery cohort were male sex.
Reporting on race, ethnicity, or other socially relevant groupings	Race and ethnicity were not collected at time of patient consent.
Population characteristics	Patients presenting for resection of sporadic vestibular schwannoma who gave consent for tumor sampling for research were included in the study. Age, sex, prior surgery, prior radiosurgery variables were collected.
Recruitment	Patients who had sporadic (non-syndromic) vestibular schwannomas who also had surgical resection of the tumor. Exclusion criteria included a history of neurofibromatosis type 2.
Ethics oversight	UCSF Institutional Review Board (#10-01318, #18-24633)

Note that full information on the approval of the study protocol must also be provided in the manuscript.

Field-specific reporting

Please select the one below that is the best fit for your research. If you are not sure, read the appropriate sections before making your selection.

☒ Life sciences ☐ Behavioural & social sciences ☐ Ecological, evolutionary & environmental sciences

For a reference copy of the document with all sections, see nature.com/documents/nr-reporting-summary-flat.pdf

Life sciences study design

All studies must disclose on these points even when the disclosure is negative.

Sample size	Clinical tumor analysis: A total of 75 clinically heterogeneous retrospective and prospective schwannomas from 67 patients who were treated from 2003 to 2020 at a single institution were obtained for analysis. Given that most profiled tumors were retrospective in nature, our sample size was limited by the inception of our schwannoma tumor banking program and associated institutional protocol. All tumors which had archived tissue for analysis were processed to maximize sample sizes.<br>Functional experiments: individual assays were performed in at least triplicates, which demonstrated high reproducibility<br>CRISPR interference screens: genome wide screens were performed in triplicate cultures, with target 500x coverage of each sgRNA in each replicate, a sample size which allows comparison of sgRNA distributions using parametric statistical testing.
Data exclusions	Patients with history of neurofibromatosis type 2 were excluded from tumor analysis.
Replication	All attempts at replication were successful. Importantly, hierarchical clustering of 125 schwannomas from an external institution also identified 2 molecular groups that were distinguished by differential DNA methylation. Functional and genomic experiments in HEI-193 cells were performed three times.
Randomization	Not a randomized study for retrospective profiling of tumors. All clinical and imaging covariates were used for recursive partitioning analysis.
Blinding	Neuroradiological, neuropathological, and experimental data analysis were performed by independent investigators with blinding of molecular groups. Group allocation was therefore blinded during sample collection.

Reporting for specific materials, systems and methods

We require information from authors about some types of materials, experimental systems and methods used in many studies. Here, indicate whether each material, system or method listed is relevant to your study. If you are not sure if a list item applies to your research, read the appropriate section before selecting a response.

Materials & experimental systems

n/a	Involved in the study
☐	☒ Antibodies
☐	☒ Eukaryotic cell lines
☒	☐ Palaeontology and archaeology
☒	☐ Animals and other organisms
☐	☒ Clinical data
☒	☐ Dual use research of concern
☒	☐ Plants

Methods

n/a	Involved in the study
☒	☐ ChIP-seq
☒	☐ Flow cytometry
☐	☒ MRI-based neuroimaging

Antibodies

Antibodies used

SOXl0 to mark schwannoma cells (API 3099, Biocare; labeling validated in schwannoma and melanoma)
Pericentrin (PA5-54109, Thermo Fisher Scientific; labeling validated by Pericentrin knockdown)
gTubulin (T5192, Sigma; labeling validated in human and chicken cells) to mark centrosomes
Acetylated Tubulin to mark cilia (T6793, Sigma; labeling validated in vertebrate and invertebrate organisms); labeled with Alexa Fluor secondary antibodies
DAPI to mark DNA (62248, Thermo Fisher Scientific);
Smoothened (ab 72130, Abeam; labeling validated by competition with immunizing peptide)
Centriolin (sc-365521, Santa Cruz Biotechnology; labeling validated using THP-1, SK-BR-3 and U-937 cell lysates) primary antibodies at 4C overnight.
CD3 (A0452, Agilent Technologies, Santa Clara, CA; labeling validated by over-expression)
CD68 (M0814, Agilent Technologies; labeling validated using human B-cell lymphoma) primary antibodies.
BCLl (RM9104R7, Thermo Fisher Scientific labeling validated using MAD109 cell lysate); Alexa Fluor 488 Anti-BrdU (Thermo Fisher Scientific, #A23210)
CyTOF antibodies are listed in Supplementary Table 5 and are reproduced below:
CD45 Biolegend 304002
CD235ab Thermo Fisher 14-9987-82
CD15 Biolegend 323002
CD3 Biolegend 300402
CD19 Biolegend 302202
CD123 Fluidigm 3143014B
CDllb Fluidigm 3144001B
CD4 Fluidigm 3145010B
CD8a Fluidigm 3146001B
CDllc Fluidigm 3147008B
CD14 Fluidigm 3148010B
CD127 Biolegend 351302
FceRI Biolegend 334602
TMEM119 Biolegend A16075D
TCRgd ATCC hybridoma/inhouse HB-9772
CD45RA Fluidigm 3153001B
SOXl0 Biolegend 847202
CD64 Biolegend 305002
PD-Ll (CD274) Fluidigm 3156026B
CD206 (MR) Biolegend 321102
CD27 Biolegend 302802
CD137 (418B) Biolegend 309802
T-bet Thermo Fisher 14-5825-82
CD80 Fluidigm 3161023B
FoxP3 Fluidigm 3162011A
CD209 (DC-SIGN) Biolegend 330102
CD45RO Fluidigm 3164007B
CD86 Biolegend 305402
CD141 (BDCA3) Biolegend 344102
CCR7 (CD197) Biolegend 353202
Ki67 Biolegend 350502
CD25 Biolegend 356102
CDlc (BDCAl) Biolegend 331502
CD68 lnvitrogen 14-0689-82
CD163 Thermo Fisher 14-1639-82
TCF-1 Biolegend 655202

	HLA-DR Fluidigm 3174001B<br>PD-1 (CD279) Biolegend 329902<br>CD56 Fluidigm 3176008B<br>CD16 Biolegend 302050
Validation	SOXl0 (labeling validated by API Biocare in human schwannoma (100%) and melanoma (96.4%) specimens)<br>Pericentrin (labeling validated by Pericentrin knockdown in MCF7 cells)<br>gTubulin (labeling validated in human and chicken cells through independent antibody verification in multiple antibodies)<br>Acetylated Tubulin (labeling validated in vertebrate and invertebrate organisms including chlamydomonas, mouse, human)<br>DAPI to mark DNA (62248, Thermo Fisher Scientific);<br>Smoothened (ab72130, Abcam; labeling validated by competition with immunizing peptide)<br>Centriolin (sc-365521, Santa Cruz Biotechnology; labeling validated using THP-1, SK-BR-3 and U-937 cell lysates) primary antibodies at 4C overnight.<br>CD3 (A0452, Agilent Technologies, Santa Clara, CA; labeling validated by over-expression)<br>CD68 (M0814, Agilent Technologies; labeling validated using human B-cell lymphoma) primary antibodies.<br>BCL1 (RM9104R7, Thermo Fisher Scientific labeling validated using MAD109 cell lysate)

Eukaryotic cell lines

Policy information about cell lines and Sex and Gender in Research

Cell line source(s)	Human Schwann cells (HSC) - ScienCell Research Laboratories<br>HEI-193 - Marco Giovannini (UCLA)<br>HEK-293T Cells - Luke Gilbert et al. 2013
Authentication	Cell lines undergo routine authentication at the UC Berkeley DNA Sequencing Facility which includes PCR amplification of 9 STR loci plus Amelogenin using Promega GenePrint 10 System, Fragment Analysis with ABI 3730XL DNA Analyzer, comprehensive data analysis with ABI Genemapper software and final verification using supplier databases including ATCC and DSMZ.
Mycoplasma contamination	All cell lines were routinely tested negative for mycoplasma.
Commonly misidentified lines<br>(See ICLAC register)	None

Clinical data

Policy information about clinical studies

All manuscripts should comply with the ICMJE guidelines for publication of clinical research and a completed CONSORT checklist must be included with all submissions.

Clinical trial registration	Not a clinical trial
Study protocol	*Note where the full trial protocol can be accessed OR if not available, explain why.*
Data collection	*Describe the settings and locales of data collection, noting the time periods of recruitment and data collection.*
Outcomes	*Describe how you pre-defined primary and secondary outcome measures and how you assessed these measures.*

Magnetic resonance imaging

Experimental design

Design type	*Indicate task or resting state; event-related or block design.*
Design specifications	*Specify the number of blocks, trials or experimental units per session and/or subject, and specify the length of each trial or block (if trials are blocked) and interval between trials.*
Behavioral performance measures	*State number and/or type of variables recorded (e.g. correct button press, response time) and what statistics were used to establish that the subjects were performing the task as expected (e.g. mean, range, and/or standard deviation across subjects).*

Acquisition

Imaging type(s)	Structural, diffusion
Field strength	3 Tesla
Sequence & imaging parameters	T2, T2 fluid attenuated inversion recovery (FLAIR), post-contrast T1 weighted images, diffusion weighted imaging (DWI), derived apparent diffusion coefficient (ADC) maps
Area of acquisition	*State whether a whole brain scan was used OR define the area of acquisition, describing how the region was determined.*

Diffusion MRI ☒ Used ☐ Not used

Parameters: 6-directional axial diffusion sequences, section thickness = 1.5 mm, number of excitations= 4, or 3-directional axial sequences, section thickness= 2.5 mm,, number of excitations = 4. No cardiac gating.

Preprocessing

Preprocessing software	*Provide detail on software version and revision number and on specific parameters (model/functions, brain extraction, segmentation, smoothing kernel size, etc.).*
Normalization	*If data were normalized/standardized, describe the approach(es): specify linear or non-linear and define image types used for transformation OR indicate that data were not normalized and explain rationale for lack of normalization.*
Normalization template	*Describe the template used for normalization/transformation, specifying subject space or group standardized space (e.g. original Talairach, MNI305, ICBM152) OR indicate that the data were not normalized.*
Noise and artifact removal	*Describe your procedure(s) for artifact and structured noise removal, specifying motion parameters, tissue signals and physiological signals (heart rate, respiration).*
Volume censoring	*Define your software and/or method and criteria for volume censoring, and state the extent of such censoring.*

Statistical modeling & inference

Model type and settings	*Specify type (mass univariate, multivariate, RSA, predictive, etc.) and describe essential details of the model at the first and second levels (e.g. fixed, random or mixed effects; drift or auto-correlation).*
Effect(s) tested	*Define precise effect in terms of the task or stimulus conditions instead of psychological concepts and indicate whether ANOVA or factorial designs were used.*

Specify type of analysis: ☐ Whole brain ☐ ROI-based ☐ Both

Statistic type for inference (See Eklund et al. 2016)	*Specify voxel-wise or cluster-wise and report all relevant parameters for cluster-wise methods.*
Correction	*Describe the type of correction and how it is obtained for multiple comparisons (e.g. FWE, FDR, permutation or Monte Carlo).*

Models & analysis

n/a	Involved in the study
☒	☐ Functional and/or effective connectivity
☒	☐ Graph analysis
☒	☐ Multivariate modeling or predictive analysis
